# Constructing Artificial SEI Layer on Lithiophilic MXene Surface for High‐Performance Lithium Metal Anodes

**DOI:** 10.1002/advs.202103930

**Published:** 2022-01-06

**Authors:** Feifei Zhao, Pengbo Zhai, Yi Wei, Zhilin Yang, Qian Chen, Jinghan Zuo, Xiaokang Gu, Yongji Gong

**Affiliations:** ^1^ School of Materials Science and Engineering Beihang University Beijing 100191 China; ^2^ College of Physics Qingdao University Qingdao 266071 China; ^3^ Beijing Key Laboratory of Electrochemical Process and Technology for Materials Beijing University of Chemical Technology Beijing 100029 China

**Keywords:** artificial solid electrolyte interfaces, dendrite‐free Li metal anodes, Li metal batteries, lithiophilicity, MXene

## Abstract

MXene has been found as a good host for lithium (Li) metal anodes because of its high specific surface area, lithiophilicity, good stability with lithium, and the in situ formed LiF protective layer. However, the formation of Li dendrites and dead Li is inevitable during long‐term cycle due to the lack of protection at the Li/electrolyte interface. Herein, a stable artificial solid electrolyte interface (SEI) is constructed on the MXene surface by using insulating g‐C_3_N_4_ layer to regulate homogeneous Li plating/stripping. The 2D/2D MXene/g‐C_3_N_4_ composite nanosheets can not only guarantee sufficient lithiophilic sites, but also protect the Li metal from continuous corrosion by electrolytes. Thus, the Ti_3_C_2_T*_x_*/g‐C_3_N_4_ electrode enables conformal Li deposition, enhanced average Coulombic efficiency (CE) of 98.4%, and longer cycle lifespan over 400 cycles with an areal capacity of 1.0 mAh cm^−2^ at 0.5 mA cm^−2^. Full cells paired with LiFePO_4_ (LFP) cathode also achieve enhanced rate capacity and cycling stability with higher capacity retention of 85.5% after 320 cycles at 0.5C. The advantages of the 2D/2D lithiophilic layer/artificial SEI layer heterostructures provide important insights into the design strategies for high‐performance and stable Li metal batteries.

## Graphical abstract

A stable artificial solid electrolyte interface is constructed on the MXene surface by using insulating g‐C_3_N_4_ layer to regulate homogeneous Li plating/stripping. The amorphous g‐C_3_N_4_ enables highly uniform artificial SEI and MXene provides sufficient lithiophilic sites for Li nucleation. The obtained Ti_3_C_2_T*_x_*/g‐C_3_N_4_ composite electrode enables conformal Li deposition, enhanced average Coulombic efficiency, and longer cycle lifespan.

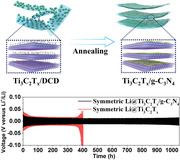

## 1 Introduction

With the increasing energy demand of electronic applications (e.g., portable electronics, electric vehicles, grid‐scale storage), the development of high‐energy‐density storage systems becomes extremely critical.^[^ ^1^, ^2^ ^]^ Lithium (Li) metal with high specific capacity (3860 mAh g^−1^) and the most negative electrochemical potential (−3.04 V vs the standard hydrogen electrode) has aroused wide concern as the ideal anode material for high‐energy‐density storage.^[^ ^3^, ^4^ ^]^ Despite the unique advantage, the practical application of rechargeable Li metal batteries still encounters some technical challenges. As the highly reactive Li reacts with the organic electrolytes spontaneously, an unstable solid electrolyte interface (SEI) is formed at the Li/electrolyte interface. The mechanically fragile SEI could constantly break and reconstruct during Li electroplating/stripping process, leading to locally enhanced Li ion flux and exacerbating the Li dendrites formation.^[^ ^5^ ^]^ Repeated growth and dissolution of the Li dendrites consume Li metal and electrolytes continuously, which results in low Coulombic efficiency (CE), poor cycling lifespan, internal short circuits, and serious safety hazards.^[^ ^6^ ^]^

In order to avoid the continuous side reactions between Li metal and electrolyte, and regulate Li deposition, a variety of strategies, including introducing lithiophilic sites, interface engineering and constructing 3D Li‐host, have been proposed to realize dendrite‐free Li metal anodes.^[^ ^7^, ^8^, ^9^, ^10^ ^]^ Introducing lithiophilic sites into the Li host could help to guide smooth Li deposition rather than the Li dendrites.^[^ ^11^, ^12^ ^]^ By adjusting the composition of electrolytes and additives^[^ ^13^ ^]^ or designing artificial protective layers for Li metal anodes to modify or replace the electrolyte‐derived SEI,^[^ ^14^, ^15^, ^16^ ^]^ it enables good regulation effects on Li deposition and suppressing Li dendrites formation effectively.^[^ ^17^, ^18^, ^19^ ^]^ The 3D hosts such as carbon‐based hosts,^[^ ^20^, ^21^ ^]^ 3D copper foam,^[^ ^22^ ^]^ and 3D Ni network^[^ ^23^ ^]^ can facilitate the transportation of Li ions or electrons, alleviate the volume changes of Li metal anodes, and enhance cycling stability effectively.^[^ ^24^ ^]^

Recently, owing to the unique physical and chemical properties, MXene as a new family of 2D materials gains particular attention and has been widely studied in energy storage devices,^[^ ^25^, ^26^ ^]^ electromagnetic interference shielding,^[^ ^27^ ^]^ catalysis,^[^ ^28^ ^]^ and so on. The general formula of MXene family is M*_n_*_+1_X*_n_* T*_x_*, where M represents early transition metals, X is carbon and/or nitrogen, and T represents the surface terminated groups such as —OH, —O, or —F.^[^ ^29^ ^]^ Among them, Ti_3_C_2_T*_x_* MXene is the most common and widely used one. MXenes usually possess excellent electrical conductivity, low Li ion diffusion barrier, as well as abundant lithiophilic functional groups on the surface, rendering it an ideal platform for Li deposition.^[^ ^30^ ^]^ Furthermore, due to the presence of inherent —F surface termination groups in MXene, a robust LiF‐containing SEI layer can be formed, achieving homogeneous Li deposition.^[^ ^31^ ^]^ MXenes as high‐performance hosts for Li metal anodes have been explored, such as 3D porous MXene aerogels,^[^ ^32^ ^]^ parallelly aligned MXene layers,^[^ ^31^ ^]^ perpendicular MXene arrays,^[^ ^33^ ^]^ and so on. Attributing to the fast transportation of Li ions, rich Li nucleation sites, and high electrical conductivity, such 3D hosts enable stable cycling performance and low overpotential even at an increased current density. Unfortunately, the nonuniform lithiophilic sites and closely stacked structure of MXene give rise to anisotropic Li nucleation. Meanwhile, the electrolyte‐derived SEI cannot protect Li metal anodes from continuous corrosion by electrolytes, finally resulting in uncontrolled dendrites growth and irreversible capacity loss. Multiple aspects, like ionic conductivity, interfacial resistance, and shape changes adaptability need to be considered to establish an efficient protective layer for Li metal anode. Consequently, a new composite containing a lithiophilic host modified by an artificial SEI layer with high homogeneity and stability is desirable for high‐performance Li metal batteries.

Herein, high‐performance Li metal anodes with the structure of 2D/2D lithiophilic layer/artificial SEI layer are designed. The obtained 2D/2D Ti_3_C_2_T*_x_*/g‐C_3_N_4_ composite electrode shows unique advantages of regulating homogeneous Li plating/stripping, protecting the Li metal from continuous corrosion by electrolytes, and curbing Li dendrites. The Ti_3_C_2_T*_x_* not only possesses the merits of good electrical conductivity, lithiophilicity, but also serves as the platform for the uniform deposition of g‐C_3_N_4_.^[^ ^32^ ^]^ Owing to the insulating nature of g‐C_3_N_4_, Li will not deposit on its surface, and the unique atomic structure provides lithium ion conduction pathway.^[^ ^34^ ^]^ Thus, the amorphous and highly insulated g‐C_3_N_4_ functions as uniform artificial SEI, regulating homogeneous Li plating/stripping on the Ti_3_C_2_T*_x_*/g‐C_3_N_4_ electrode.^[^ ^24^, ^35^ ^]^ Benefiting from these advantages of 2D/2D lithiophilic/insulating composite nanosheets, the Ti_3_C_2_T*_x_*/g‐C_3_N_4_ composite electrode delivers a high average CE of 98.4% and good stability over 400 cycles with an areal capacity of 1.0 mAh cm^−2^ at 0.5 mA cm^−2^, much better than that of pristine Ti_3_C_2_T*_x_* electrode. When the Li deposited Ti_3_C_2_T*_x_*/g‐C_3_N_4_ electrode is paired with LiFePO_4_ (LFP) cathode, the full cell exhibits an improved rate capability and stable cycling performance with higher capacity retention of 85.5% after 320 cycles at 0.5C.

## 2 Results and Discussion

**Figure 1:**
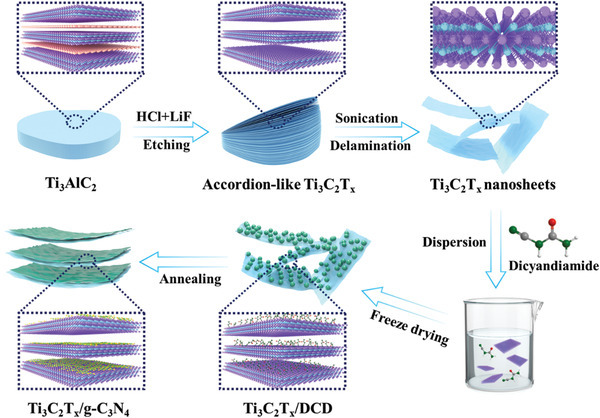
Schematic illustration of the fabrication process of Ti_3_C_2_T*_x_*/g‐C_3_N_4_ hybrid.

**Figure** **1** schematically illustrates the fabrication process of Ti_3_C_2_T*_x_*/g‐C_3_N_4_ hybrid. Ti_3_C_2_T*_x_*/g‐C_3_N_4_ hybrid was prepared through the processes of self‐assembly and in situ calcination reaction. First, Ti_3_C_2_T*_x_* was synthesized using the minimal intensive layer delamination (MILD) method, which was based on selectively removing Al layer from Ti_3_AlC_2_ with LiF/HCl etchant.^[^ ^29^ ^]^ After etching, the Ti_3_C_2_T*_x_* was bounded with various surface termination groups like —OH, —O, and —F. The obtained MXene nanosheets and dicyanamide (DCD) formed a homogeneous solution via sonication. During the self‐assembly process, DCD monomers were tightly adsorbed on the surface of Ti_3_C_2_T*_x_* due to the surface functional groups induced interaction (e.g., hydrogen bonding).^[^ ^36^ ^]^ The obtained Ti_3_C_2_T*_x_*/DCD (Figure S1, Supporting Information) composite after freeze‐drying was then annealed at a proper temperature, during which process DCD was in situ condensed into g‐C_3_N_4_, enabling uniform g‐C_3_N_4_ coating layers on the surface of Ti_3_C_2_T*_x_*. The pyrolysis process of Ti_3_C_2_T*_x_*/DCD precursor can also prevent Ti_3_C_2_T*_x_* nanosheets from restacking. The intimate coupling between Ti_3_C_2_T*_x_* and g‐C_3_N_4_ is beneficial for the structural stability. Thus, the ultrathin 2D/2D Ti_3_C_2_T*_x_*/g‐C_3_N_4_ nanosheets heterojunction was obtained, allowing for enhanced ionic/electronic transport in the porous interconnected network structure.

**Figure 2:**
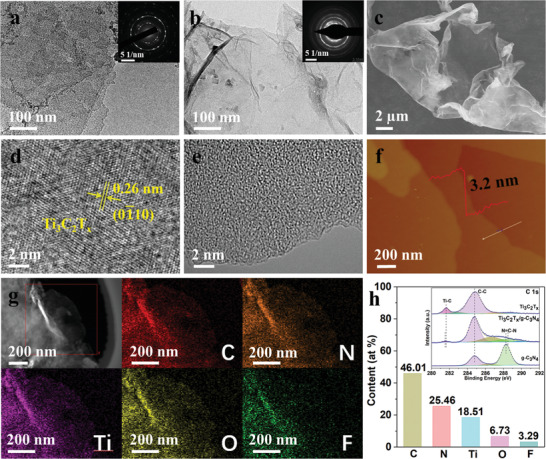
Morphology and chemical composition characterizations. TEM images of a) bare Ti_3_C_2_T*_x_* and b) Ti_3_C_2_T*_x_*/g‐C_3_N_4_ hybrid. The inset images in panels (a) and (b) are the corresponding SAED patterns. c) SEM image of Ti_3_C_2_T*_x_*/g‐C_3_N_4_. High‐resolution TEM images of d) Ti_3_C_2_T*_x_* and e) Ti_3_C_2_T*_x_*/g‐C_3_N_4_. f) AFM image and thickness profile of Ti_3_C_2_T*_x_*/g‐C_3_N_4_. g) HAADF‐STEM image of Ti_3_C_2_T*_x_*/g‐C_3_N_4_ and the corresponding elemental mapping images of C, N, Ti, O, and F elements, respectively. h) Element contents obtained by XPS measurement in Ti_3_C_2_T*_x_*/g‐C_3_N_4_. The inset shows high‐resolution C 1s spectra of the samples.

Morphology and chemical composition characterizations of the samples are shown in **Figure** **2**. The transmission electron microscopy (TEM; Figure 2a) and scanning electron microscopy (SEM; Figure S2a, Supporting Information) images of bare Ti_3_C_2_T*_x_* present the ultrathin nanosheet morphology with ultrathin thickness (Figure S2b, Supporting Information). The selected area electron diffraction (SAED) pattern indicates the polycrystalline structure of Ti_3_C_2_T*_x_* nanosheets (inset of Figure 2a). The as‐prepared Ti_3_C_2_T*_x_* is modified by g‐C_3_N_4_ and the Ti_3_C_2_T*_x_*/g‐C_3_N_4_ hybrid shows a crinkled sheet‐like morphology with a thickness of 3.2 nm (Figure 2b,c,f). The lattice spacing of 0.26 nm observed by high‐resolution TEM (HRTEM) corresponds to the (010) plane of Ti_3_C_2_T*_x_* (Figure 2d).^[^ ^37^ ^]^ The HRTEM image of Ti_3_C_2_T*_x_*/g‐C_3_N_4_ (Figure 2e) shows an amorphous feature, as the same with the HRTEM image of g‐C_3_N_4_ (Figure S3b, Supporting Information), indicating the heterostructures generated by stacking multilayered g‐C_3_N_4_ on the surface of Ti_3_C_2_T*_x_*. According to the high‐angle annular dark‐field scanning TEM (HAADF‐STEM) image and the corresponding elemental mapping images of Ti_3_C_2_T*_x_*/g‐C_3_N_4_ (Figure 2g), the C, N, Ti, O, and F elements homogeneously distribute over the sample, further confirming the Ti_3_C_2_T*_x_* is uniformly coated by g‐C_3_N_4_ layers.

The phase structures of as‐prepared samples were investigated by X‐ray diffraction (XRD) characterization (Figure S4, Supporting Information). After etching treatment, the intense diffraction peak of (104) planes (2*θ* = 39°) in Ti_3_AlC_2_ disappears and both the peaks of (002) and (004) planes shift to lower degrees, suggesting the successful removal of Al layer from Ti_3_AlC_2_ and the synthesis of 2D layered Ti_3_C_2_T*_x_*.^[^ ^38^, ^39^ ^]^ The peak of (002) planes in the XRD pattern of Ti_3_C_2_T*_x_*/g‐C_3_N_4_ shifts left compared with that of pristine Ti_3_C_2_T*_x_*, suggesting that the layer spacing of Ti_3_C_2_T*_x_* further expands owing to the coating of g‐C_3_N_4_.^[^ ^40^ ^]^ The XRD pattern of g‐C_3_N_4_ displays two typical diffraction peaks of (100) planes (2*θ* = 12.8°) and (002) planes (2*θ* = 27.2°), corresponding to the in‐plane packing of structural motifs and the interplanar stacking of conjugated aromatic systems, respectively.^[^ ^41^ ^]^ However, the diffraction peaks assigned to g‐C_3_N_4_ cannot be observed in Ti_3_C_2_T*_x_*/g‐C_3_N_4_, indicating that no bulk g‐C_3_N_4_ is produced and the Ti_3_C_2_T*_x_* maintains its crystalline structure after hybridization.^[^ ^36^ ^]^ The presence of g‐C_3_N_4_ on the surface of Ti_3_C_2_T*_x_* is further revealed by Fourier transform infrared spectroscopy (FTIR). FTIR spectrum of Ti_3_C_2_T*_x_*/g‐C_3_N_4_ (Figure S5, Supporting Information) presents the typical vibration peaks of g‐C_3_N_4_, that are the peaks at 810 cm^−1^ arising from the symmetric stretching vibration of triazine units, as well as the peaks in the range of 1200 to 1650 cm^−1^ attributing to the stretching vibration of C—N and C═N heterocycles.^[^ ^42^ ^]^ In the FTIR spectra of Ti_3_C_2_T*_x_* and Ti_3_C_2_T*_x_*/g‐C_3_N_4_, which contain surface termination groups like —OH, —O, and —F, the obvious broad band at around 3430 cm^−1^ ascribes to the —OH stretching vibration.^[^ ^43^ ^]^ The above results confirm the successful hybridization of Ti_3_C_2_T*_x_* and g‐C_3_N_4_ and the structures of Ti_3_C_2_T*_x_* and g‐C_3_N_4_ are well maintained after hybridization. In addition, N_2_ adsorption–desorption measurements were conducted to investigate the specific surface area and structure of the samples (Figure S6, Supporting Information). The specific surface areas of Ti_3_C_2_T*_x_*, g‐C_3_N_4_, and Ti_3_C_2_T*_x_*/g‐C_3_N_4_ are 15.2, 11.0, and 24.9 m^2^ g^−1^, respectively. All the samples possess type‐IV isotherms with obvious hysteresis loops, indicating the mesoporous characteristics.

Figure 2h shows the element contents obtained by X‐ray photoelectron spectroscopy (XPS) measurement in Ti_3_C_2_T*_x_*/g‐C_3_N_4_, in which the C, N, Ti, O, and F contents are determined to be 46.01, 25.46, 18.51, 6.73, and 3.29 at%, respectively (Figure S7, Supporting Information). The C 1s spectrum of g‐C_3_N_4_ displays two obvious peaks at 284.8 and 288.3 eV, attributing to the C—C bonds and characteristic N═C—N bonds in triazine rings, respectively.^[^ ^44^ ^]^ In the C 1s spectrum of Ti_3_C_2_T*_x_*/g‐C_3_N_4_, the peak at 281.5 eV ascribes to Ti—C bonds of Ti_3_C_2_T*_x_*,^[^ ^45^ ^]^ as well as the peak at 288.5 eV ascribes to N═C—N bonds of g‐C_3_N_4_. It can be observed that the peak corresponding to Ti—C bonds in Ti_3_C_2_T*_x_*/g‐C_3_N_4_ shows a slightly shift of 0.1 eV to lower binding energy region in comparison with that of Ti_3_C_2_T*_x_*. In contrast, the peak belonged to N═C—N bonds in Ti_3_C_2_T*_x_*/g‐C_3_N_4_ shifts 0.2 eV to higher binding energy region compared to that of g‐C_3_N_4_. The slight binding energy shift implies that the interaction between Ti_3_C_2_T*_x_* and g‐C_3_N_4_ promotes the interfacial charge transfer.^[^ ^36^ ^]^ The high resolution Ti 2p spectra of Ti_3_C_2_T*_x_* and Ti_3_C_2_T*_x_*/g‐C_3_N_4_ composite could be deconvoluted into six components, including the peaks at 461.0 and 455.0 eV ascribed to Ti–C 2p_1/2_ and Ti–C 2p_3/2_, the peaks at 464.7 and 459.1 eV ascribed to Ti–O 2p_1/2_ and Ti–O 2p_3/2_, respectively. Besides, the peaks at 462.5 and 456.3 eV are ascribed to Ti–X (Ti^2+^), which corresponds to substoichiometric titanium carbide or titanium oxycarbides.^[^ ^46^ ^]^ In the high‐resolution N 1s spectrum of Ti_3_C_2_T*_x_*/g‐C_3_N_4_, three peaks located at 398.6, 400.1, and 400.9 eV, correspond to pyridinic N (C—N═C), graphitic N (N—(C)_3_), and amino groups (C—N—H), respectively.^[^ ^47^, ^48^ ^]^ The peak at 396.6 eV is assigned to Ti—N bond, implying the strong combination between Ti_3_C_2_T*_x_* and g‐C_3_N_4_. The Ti_3_C_2_T*_x_*/g‐C_3_N_4_ hybrid was optimized by tuning the addition amount of DCD in the sample preparation process. Thus, Ti_3_C_2_T*_x_*/g‐C_3_N_4_ composites with different g‐C_3_N_4_ content were prepared (Figure S8, Supporting Information), which was denoted as Ti_3_C_2_T*_x_*/g‐C_3_N_4_‐3, Ti_3_C_2_T*_x_*/g‐C_3_N_4_‐5, Ti_3_C_2_T*_x_*/g‐C_3_N_4_‐7, and Ti_3_C_2_T*_x_*/g‐C_3_N_4_‐9, respectively. The N content in Ti_3_C_2_T*_x_*/g‐C_3_N_4_ composites increases from 16.74 to 35.79 at%.

**Figure 3:**
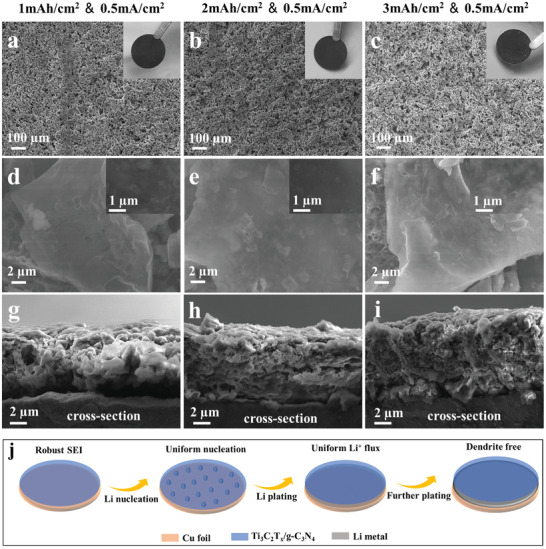
Morphology characterizations of Li deposition on Ti_3_C_2_T*_x_*/g‐C_3_N_4_ electrodes. SEM images of Li metal deposited on Ti_3_C_2_T*_x_*/g‐C_3_N_4_ electrodes after plating: a,d) 1 mAh cm^−2^, b,e) 2 mAh cm^−2^, and c,f) 3 mAh cm^−2^. The inset images in panels (a)–(c) are photographs of the corresponding electrodes. Insets in panels (d)–(f): SEM images of the surface of Li‐deposited Ti_3_C_2_T*_x_*/g‐C_3_N_4_. Cross‐section SEM images of Li metal deposited on Ti_3_C_2_T*_x_*/g‐C_3_N_4_ electrodes after plating: g) 1 mAh cm^−2^, h) 2 mAh cm^−2^, and i) 3 mAh cm^−2^. j) Schematic illustration of the Li plating behaviors on Ti_3_C_2_T*_x_*/g‐C_3_N_4_ electrode.

To investigate the effect of the Ti_3_C_2_T*_x_*/g‐C_3_N_4_ composites on the Li deposition behavior, different amounts of Li were deposited on Ti_3_C_2_T*_x_*/g‐C_3_N_4_ and Ti_3_C_2_T*_x_* electrodes. **Figure** **3**a shows the SEM image of Ti_3_C_2_T*_x_*/g‐C_3_N_4_ electrode after plating 1 mAh cm^−2^ of Li at a current density of 0.5 mA cm^−2^. The Ti_3_C_2_T*_x_*/g‐C_3_N_4_ electrode displays smooth appearance without Li dendrites formation. The surface of Li‐deposited Ti_3_C_2_T*_x_*/g‐C_3_N_4_ exhibits a uniform morphology and no large Li agglomeration can be observed (Figure 3d). When the Li plating capacity was increased to 2 and 3 mAh cm^−2^, respectively, the electrode did not show obvious change (Figure 3b,c). Likewise, the surface of Ti_3_C_2_T*_x_*/g‐C_3_N_4_ remained flat during Li plating process (Figure 3e,f), indicating the uniform Li deposition for the Ti_3_C_2_T*_x_*/g‐C_3_N_4_ electrode. What's more, when the Li metal was stripped, the surface of Ti_3_C_2_T*_x_*/g‐C_3_N_4_ electrode still kept smooth with the absence of residual Li particles (Figure S9, Supporting Information). Even after 50 cycles of Li plating/stripping (Figures S10–S12, Supporting Information), the Ti_3_C_2_T*_x_*/g‐C_3_N_4_ electrode retained the original overall morphology, indicating the good structure stability ensured by the artificial SEI layer. Cross‐sections of Li metal deposited Ti_3_C_2_T*_x_*/g‐C_3_N_4_ electrodes were characterized to reveal the electrode changes (Figure 3g–i). With the improvement of Li plating capacity, the electrode became more compact. The overall morphology of the electrode remained intact, and no Li dendrites can be found, indicating the uniform and conformal Li deposition on Ti_3_C_2_T*_x_*/g‐C_3_N_4_ electrode. The overall thickness of the electrode only increased 15% with Li plating capacity of 3 mAh cm^−2^ compared to the initial thickness, which is important for the overall stability of the batteries. According to the corresponding elemental mappings of Ti_3_C_2_T*_x_*/g‐C_3_N_4_ electrode (Figure S13, Supporting Information), the C, N, Ti, and O elements homogeneously distribute over the cross‐section of Ti_3_C_2_T*_x_*/g‐C_3_N_4_. Consequently, for the Li‐deposited Ti_3_C_2_T*_x_*/g‐C_3_N_4_ electrode, Ti_3_C_2_T*_x_* and g‐C_3_N_4_ are uniformly distributed over the sample, and Li is homogeneously deposited in the 3D lithiophilic host. Figure 3j illustrates the Li plating behaviors on Ti_3_C_2_T*_x_*/g‐C_3_N_4_ electrode. For Ti_3_C_2_T*_x_*/g‐C_3_N_4_ electrode, the Ti_3_C_2_T*_x_* layers provide sufficient lithiophilic sites for Li nucleation. Importantly, due to the insulating nature of g‐C_3_N_4_, Li will not deposit on its surface, and the unique atomic structure provides lithium ion conduction pathway.^[^ ^24^, ^35^ ^]^ The amorphous state of g‐C_3_N_4_ enables the high homogeneity of artificial SEI film and conformal Li deposition is achieved in the plating process. The 3D interconnected network structure of Ti_3_C_2_T*_x_*/g‐C_3_N_4_ possessing good structural stability can accommodate the volume changes effectively during Li plating/stripping cycling.

**Figure 4:**
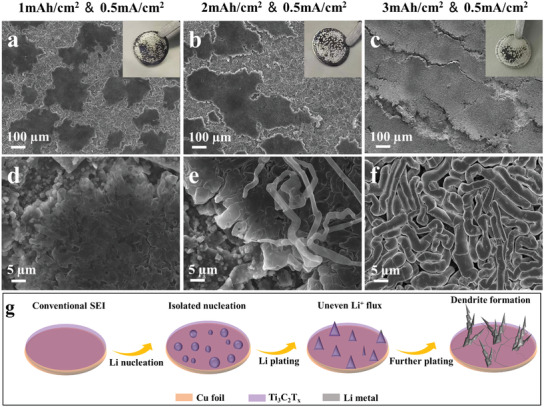
a–f) Morphology characterizations of Li deposition on Ti_3_C_2_T*_x_* electrodes. SEM images of Li metal deposited on Ti_3_C_2_T*_x_* electrodes after plating: a,d) 1 mAh cm^−2^, b,e) 2 mAh cm^−2^, and c,f) 3 mAh cm^−2^. The inset images in panels (a)–(c) are photographs of the corresponding electrodes. g) Schematic illustration of the Li plating behaviors on Ti_3_C_2_T*_x_* electrode.

For comparison, the morphology evolution processes of Li plating/stripping on Ti_3_C_2_T*_x_* electrodes are shown in **Figure** **4**a–f. After plating 1 mAh cm^−2^ of Li, the Ti_3_C_2_T*_x_* electrode presents rough surface state, large amounts of metallic Li accumulating on the upper surface of Ti_3_C_2_T*_x_* (Figure 4a). The enlarged view of image demonstrates the mossy‐like Li deposition with undulating edges (Figure 4d). Further increasing the Li deposition capacity to 2 mAh cm^−2^, the uneven Li deposition and dendrite growth can be observed on the electrode (Figure 4b,e), which may pierce the separator and result in short circuit. When the Li plating capacity reached 3 mAh cm^−2^, the number and dimension of Li dendrites were obviously increased, and the surface of Ti_3_C_2_T*_x_* was mostly covered by Li dendrites (Figure 4c,f). The surface changes can also be reflected by photographs of the corresponding electrodes. In addition, when the Li metal was stripped, numerous nano‐ or microparticles remained on the surface of Ti_3_C_2_T*_x_* electrode (Figure S14, Supporting Information). After 50 cycles of Li plating/stripping (Figure S15, Supporting Information), the surface became very rough, on which severe structural damages and dead Li can be observed, indicating the poor SEI stability. Figure 4g illustrates the Li plating behaviors on Ti_3_C_2_T*_x_* electrode. For bare Ti_3_C_2_T*_x_*, the lack of a stable SEI gives rise to anisotropic and scattered Li nucleation. During the Li plating process, Li deposits on the Ti_3_C_2_T*_x_* surface unevenly, finally resulting in dendrites formation and SEI cracks.

**Figure 5:**
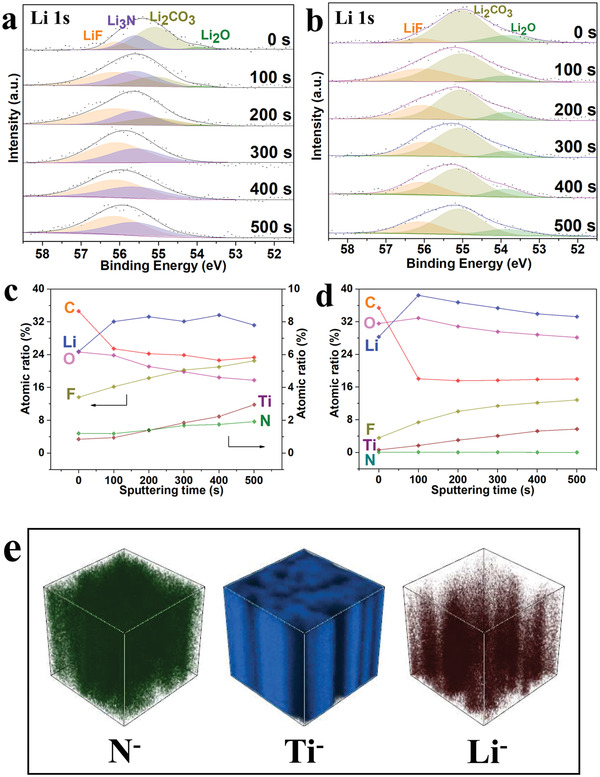
Li 1s XPS depth profiles of a) Ti_3_C_2_T*_x_*/g‐C_3_N_4_ and b) Ti_3_C_2_T*_x_* electrodes after depositing 1 mAh cm^−2^ of Li at 0.5 mA cm^−2^. The atomic composition ratios of c) Ti_3_C_2_T*_x_*/g‐C_3_N_4_ and d) Ti_3_C_2_T*_x_* electrodes at different sputtering times after depositing 1 mAh cm^−2^ of Li at 0.5 mA cm^−2^. The sputtering time increased from 0 to 500 s. e) A 3D view of the distribution of N^−^, Ti^−^, and Li^−^ in the Li deposited Ti_3_C_2_T*_x_*/g‐C_3_N_4_ electrode from TOF‐SIMS test.

The Li plating/stripping behaviors are closely related to the properties of SEI. XPS depth analysis with argon ion (Ar^+^) sputtering on Ti_3_C_2_T*_x_*/g‐C_3_N_4_ electrodes after depositing 1 mAh cm^−2^ of Li at 0.5 mA cm^−2^ is performed to disclose the composition of SEI. As is shown in the Li 1s spectra (**Figure** **5**a) of Li deposited Ti_3_C_2_T*_x_*/g‐C_3_N_4_, in the initial stage, LiF (56.1 eV), Li_3_N (55.6 eV), Li_2_CO_3_ (55.1 eV), and trace Li_2_O (53.9 eV) exist in the outer layer of SEI.^[^ ^24^, ^49^ ^]^ With the increase of Ar^+^ sputtering time, the Li_2_O peak vanishes and the proportion of Li_2_CO_3_ declines sharply, suggesting that the electrolyte decomposition is curbed. The presence of Li_3_N in the SEI layer can effectively boost the rapid transport of Li ion across the electrode surface.^[^ ^35^, ^50^ ^]^ Li_3_N, as well as LiF deriving from Li interaction with —F surface termination groups of Ti_3_C_2_T*_x_*, become the main component in the inner layer of SEI. It is generally recognized that LiF with high surface energy is beneficial for fast Li ion diffusion and homogeneous Li deposition.^[^ ^51^ ^]^ In addition, in the N 1s spectra (Figure S16, Supporting Information) of Li deposited Ti_3_C_2_T*_x_*/g‐C_3_N_4_, there exist five N species with the binding energy of 400.9 eV (C—N—H), 400.1 eV (N—(C)_3_), 399.2 eV (Li—N), 398.6 eV (C—N═C), and 396.6 eV (Ti—N), respectively.^[^ ^24^, ^46^ ^]^ The Li—N bond is mainly attributed to the strong Li interaction with N species in g‐C_3_N_4_.^[^ ^35^ ^]^ Among them, the C—N═C and N—(C)_3_ characteristic peaks of g‐C_3_N_4_ are dominant, indicating the structure stability of g‐C_3_N_4_ after lithium plating. The abundant N species in g‐C_3_N_4_ can regulate homogeneous Li ion flux on the surface of electrode and lower the nucleation overpotential.^[^ ^34^ ^]^ According to the atomic composition ratios of Li deposited Ti_3_C_2_T*_x_*/g‐C_3_N_4_ electrode at different sputtering time (Figure 5c), Li content increases in the initial sputtering stage, then remains almost constant with further sputtering, while the O content decreases quickly. Meanwhile, the content of Ti and N increase steadily. These results suggest that g‐C_3_N_4_ layer participates in the formation of SEI, curb the electrolyte decomposition, and regulate homogeneous Li plating/stripping. Moreover, the composition of SEI is further studied with time‐of‐flight secondary‐ion mass spectroscopy (TOF‐SIMS) characterization (Figure S17, Supporting Information). The high signal intensity of LiF^−^ and Li_3_N^−^ fragments proves the presence of LiF and Li_3_N in SEI. The Li_2_CO_3_^−^ fragment appears as weak signal and the Li_2_O^−^ fragment is hardly detected, indicating the low Li_2_CO_3_ content and trace Li_2_O in SEI. The signal intensity of Li^−^ fragment is weak initially, then increases gradually with further sputtering. Figure 5e shows the 3D view of the individual distribution of N^−^, Ti^−^, and Li^−^ from TOF‐SIMS test. N^−^ fragment is uniformly distributed in the 3D view. Ti^−^ fragment represents Ti_3_C_2_T*_x_*, which serves as a 3D host for Li. Li^−^ fragment is mainly distributed in the 3D interconnected network constructed by Ti_3_C_2_T*_x_*/g‐C_3_N_4_.

In comparison, the Li 1s XPS depth profiles (Figure 5b) of Li deposited Ti_3_C_2_T*_x_* indicate that the outer layer of SEI contains LiF, Li_2_O, and Li_2_CO_3_, which mainly derive from the electrolyte decomposition.^[^ ^52^ ^]^ Further sputtering, the proportion of LiF increases and then remains relatively stable resulting from the presence of F‐terminated Ti_3_C_2_T*_x_*. Moreover, a certain amount of Li_2_O was detected, which can be attributed to electrolyte decomposition, as well as the oxidation of deposited Li metal in sample preparation process. During the process of sputtering, Li_2_CO_3_ always occupies the dominant percent in the SEI layer, indicating the severe electrolyte decomposition and lithium consumption on the surface of Ti_3_C_2_T*_x_* electrode. According to the atomic composition ratios of Li deposited Ti_3_C_2_T*_x_* electrode at different sputtering time (Figure 5d), Li, C, and O always keep higher content in the SEI, and no N content can be detected, which further confirm the large amount of electrolyte decomposition and Li dendrites formation.

**Figure 6:**
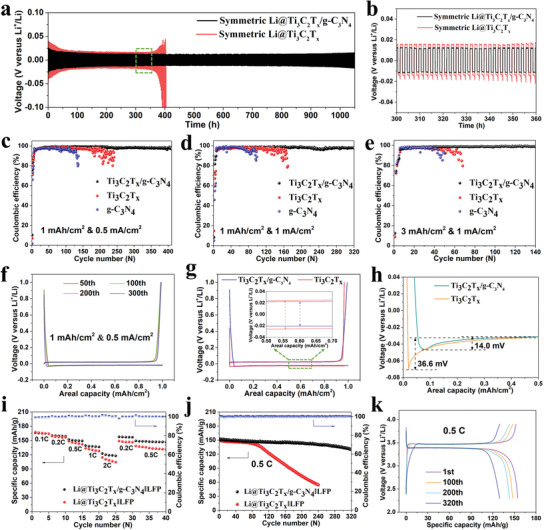
Electrochemical performance of the symmetric cells based on Li@Ti_3_C_2_T*_x_*/g‐C_3_N_4_ and Li@Ti_3_C_2_T*_x_* electrodes: a) Voltage–time profiles of Li plating/stripping in different symmetric cells with the capacity of 0.5 mAh cm^−2^ at 0.5 mA cm^−2^. b) Detailed voltage profiles for selective cycles in panel (a). Electrochemical performance of the half cells: Coulombic efficiency of galvanostatic Li plating/stripping cycling on Ti_3_C_2_T*_x_*/g‐C_3_N_4_, Ti_3_C_2_T*_x_*, and g‐C_3_N_4_ electrodes with the capacity of c) 1 mAh cm^−2^ at 0.5 mA cm^−2^, d) 1 mAh cm^−2^ at 1 mA cm^−2^, and e) 3 mAh cm^−2^ at 1 mA cm^−2^. f) Voltage profiles of the 50th, 100th, 200th, and 300th cycling on Ti_3_C_2_T*_x_*/g‐C_3_N_4_ electrode with a capacity of 1 mAh cm^−2^ at 0.5 mA cm^−2^, respectively. g) Voltage profiles of the 50th cycling on Ti_3_C_2_T*_x_*/g‐C_3_N_4_ and Ti_3_C_2_T*_x_* electrodes with a capacity of 1 mAh cm^−2^ at 0.5 mA cm^−2^. h) Voltage–areal capacity curves during Li nucleation process on Ti_3_C_2_T*_x_*/g‐C_3_N_4_ and Ti_3_C_2_T*_x_* electrodes at 0.5 mA cm^−2^. Electrochemical performance of Li@Ti_3_C_2_T*_x_*/g‐C_3_N_4_||LFP and Li@Ti_3_C_2_T*_x_* ||LFP full cells: i) rate performance, j) cycling performance at 0.5C, and k) charge/discharge curves during cycling of Li@Ti_3_C_2_T*_x_*/g‐C_3_N_4_||LFP. The areal capacity of LFP was 0.64 mAh cm^−2^ and the N/P ratio was 2.5.

Electrochemical performance of the symmetric cells based on Li@Ti_3_C_2_T*_x_*/g‐C_3_N_4_ and Li@Ti_3_C_2_T*_x_* electrodes was tested to explore the Li plating/stripping behaviors on different electrodes. As shown in **Figure** **6**a,b, when the cycling capacity was 0.5 mAh cm^−2^ at a current density of 0.5 mA cm^−2^, the symmetrical Li@Ti_3_C_2_T*_x_*/g‐C_3_N_4_ cell exhibited stable cycling for 1050 h with a low overpotential of about 12 mV, indicating the unique advantages of uniform nucleation and homogeneous Li plating/stripping in the 2D/2D Ti_3_C_2_T*_x_*/g‐C_3_N_4_ composite electrode. In comparison, the symmetrical Li@Ti_3_C_2_T*_x_* cell exhibited a higher overpotential and cycled less than 400 h before fluctuation, which can be attributed to the uneven Li nucleation and uncontrolled dendrites growth. Furthermore, half cells were assembled with working electrodes pairing Li foils as the counter electrodes. Figure 6c–e shows the CE of galvanostatic charge–discharge cycles at different Li deposition capacities and current densities. As shown in Figure 6c, when the cycling capacity was 1 mAh cm^−2^ at a current density of 0.5 mA cm^−2^, Ti_3_C_2_T*_x_*/g‐C_3_N_4_ electrode exhibited a high average CE of 98.4% for more than 400 cycles. In comparison, for Ti_3_C_2_T*_x_* electrode, the average CE was 97.6% for the previous 120 cycles, then decayed gradually and reduced to below 90% after the 200th cycle. The relatively low CE in the initial several cycles can be attributed to the initial activation of g‐C_3_N_4_ artificial SEI layer and the reduction of oxygen‐containing species and fluorine termination groups on the Ti_3_C_2_T*_x_*/g‐C_3_N_4_ electrode (or Ti_3_C_2_T*_x_* electrode), which might induce the sluggish Li ions transport.^[^ ^34^ ^]^ When the current density was increased to 1.0 mA cm^−2^ with the same capacity (Figure 6d), the average CE of 98.0% was achieved for 320 cycles within the Ti_3_C_2_T*_x_*/g‐C_3_N_4_ electrode. However, in the same condition, the stable CE of Ti_3_C_2_T*_x_* electrode could be just maintained for 120 cycles followed by fluctuating. Even cycling with a high capacity of 3 mAh cm^−2^ at a current density of 1 mA cm^−2^, the CE of Ti_3_C_2_T*_x_*/g‐C_3_N_4_ electrode could remain relatively stable at 98.5% for 140 cycles, while the CE of Ti_3_C_2_T*_x_* electrode dropped down significantly after 45 cycles (Figure 6e). Moreover, for the Ti_3_C_2_T*_x_*/g‐C_3_N_4_ electrode, high average CE of 98.6% can still be obtained for 150 cycles at an elevated current density of 2 mA cm^−2^ with a capacity of 2 mAh cm^−2^ (Figure S18, Supporting Information). When the cycling capacity was 3 mAh cm^−2^ at a current density of 3 mA cm^−2^, Ti_3_C_2_T*_x_*/g‐C_3_N_4_ electrode exhibited an average CE of 98.2% for 120 cycles (Figure S19, Supporting Information). The irreversible capacity loss can be ascribed to the SEI recovery and dead Li formation on the surface of the electrode material.^[^ ^53^ ^]^

The Li plating/stripping behaviors on different electrodes were also investigated by the charge/discharge curves. As shown in Figure 6f, the voltage profiles of different cycles on Ti_3_C_2_T*_x_*/g‐C_3_N_4_ electrode are almost coincident, indicating the excellent reversibility of the Li plating/stripping behaviors on Ti_3_C_2_T*_x_*/g‐C_3_N_4_ electrode consistent with the high CE retention. When comparing the voltage profiles of the same cycle, it is obvious that the voltage hysteresis of Ti_3_C_2_T*_x_*/g‐C_3_N_4_ electrode is smaller than that of the Ti_3_C_2_T*_x_* electrode, which can be attributing to the stable artificial SEI layer and lower interface impedance of Ti_3_C_2_T*_x_*/g‐C_3_N_4_ electrode (Figure 6g). Figure 6h shows the nucleation overpotential of Li on both electrodes. The value of Ti_3_C_2_T*_x_*/g‐C_3_N_4_ electrode is 14.0 mV, which is much lower than that of the Ti_3_C_2_T*_x_* electrode (36.6 mV), indicating the smaller Li deposition barrier on Ti_3_C_2_T*_x_*/g‐C_3_N_4_ electrode. In addition, electrochemical impedance spectroscopy (EIS) measurements at different cycles were performed to explore the charge transfer properties on electrode/electrolyte interface (Figure S20, Supporting Information). The Ti_3_C_2_T*_x_* electrode exhibits increasing SEI resistance with cycling, the same with the charge transfer resistance after the initial cycles (Table S1, Supporting Information), which is ascribed to the side reactions between electrolyte and the deposited Li metal. Comparing with the Ti_3_C_2_T*_x_* electrode, the SEI resistance and charge transfer resistance on Ti_3_C_2_T*_x_*/g‐C_3_N_4_ electrode are much smaller and keep stable, demonstrating the high ionic conductivity of artificial SEI layer and the improved interfacial stability.

The electrochemical performance of Ti_3_C_2_T*_x_*/g‐C_3_N_4_ electrode was further tested in full cell assembled with commercial LFP as the cathode, and Ti_3_C_2_T*_x_*/g‐C_3_N_4_ electrode with Li deposition (denoted as Li@Ti_3_C_2_T*_x_*/g‐C_3_N_4_) as anode. The areal capacity of LFP was 0.64 mAh cm^−2^ and the N/P ratio was 2.5 (Figure S21, Supporting Information). As shown in Figure 6i, the Li@Ti_3_C_2_T*_x_*/g‐C_3_N_4_||LFP full cell exhibits a good rate capability (121 mAh g^−1^ at 2.0C). By comparison, though the Li@Ti_3_C_2_T*_x_* ||LFP full cell tested in the same conditions exhibits comparable capacity at low current density, the capacity fades quickly with the increase of current density. The Li@Ti_3_C_2_T*_x_*/g‐C_3_N_4_||LFP full cell also has improved cycling stability with higher capacity retention of 85.5% after 320 cycles at 0.5C (Figure 6j). Figure 6k shows the steady charge/discharge curves without obvious overpotential increase during cycling of Li@Ti_3_C_2_T*_x_*/g‐C_3_N_4_||LFP, indicating the excellent interfacial stability. In addition, the areal capacity of LFP was 0.64 mAh cm^−2^ and the N/P ratio was reduced to 0.5. The Li@Ti_3_C_2_T*_x_*/g‐C_3_N_4_||LFP full cell maintains a cycling stability for 170 cycles at 1C, and the capacity retention is 85.7% (Figure S22, Supporting Information). In comparison, the Li@Ti_3_C_2_T*_x_* ||LFP full cell shows rapid capacity decay and the capacity retention is only 47.6% after 170 cycles.

The electrochemical performance of full cells paired with high areal loading of LFP was further tested. As shown in Figure S23 in the Supporting Information, the areal capacity of LFP was increased to 2.2 mAh cm^−2^ and the N/P ratio was 1.0. For the Li@Ti_3_C_2_T*_x_*/g‐C_3_N_4_||LFP full cell, a stable cycling of 150 cycles at 1C is achieved with a capacity retention of 83.8%. Moreover, when paired with a high‐energy cathode of NCM811 (areal capacity of 1.2 mAh cm^−2^), the Li@Ti_3_C_2_T*_x_*/g‐C_3_N_4_||NCM full cell delivers an initial specific capacity of 167.2 mAh g^−1^ at 0.3C, and keeps stable cycling for 110 cycles with a capacity retention of 81.2% (Figure S24, Supporting Information). These results suggest that Ti_3_C_2_T*_x_*/g‐C_3_N_4_ composite electrode possesses unique advantages for high‐performance and stable Li metal batteries.

## 3 Conclusion

In conclusion, based on the 2D/2D lithiophilic layer/artificial SEI layer heterostructures, a stable artificial SEI is constructed on the MXene surface by using insulating g‐C_3_N_4_ layer to regulate homogeneous Li plating/stripping. The amorphous g‐C_3_N_4_ enables high uniformity of artificial SEI film and MXene provides sufficient lithiophilic sites for Li nucleation. The obtained 2D/2D Ti_3_C_2_T*_x_*/g‐C_3_N_4_ composite electrode shows unique advantages of regulating homogeneous Li plating/stripping, protecting the Li metal from continuous corrosion by electrolytes, and curbing Li dendrites. As a result, the Ti_3_C_2_T*_x_*/g‐C_3_N_4_ composite electrode exhibits conformal Li deposition and stable cycling over 400 cycles with an areal capacity of 1.0 mAh cm^−2^ at 0.5 mA cm^−2^. Full cells paired with LFP cathode also achieve enhanced rate capacity and cycling stability with higher capacity retention of 85.5% after 320 cycles at 0.5C. The advantages of 2D/2D lithiophilic/insulating heterostructures enlighten the design strategies of stable Li metal anodes and promote the practical application of Li metal batteries.

## 4 Experimental Section

### Synthesis of Ti_3_C_2_T*_x_* Nanosheets

Ti_3_C_2_T*_x_* nanosheets were synthesized using the MILD method. Typically, 0.8 g of LiF was added into 10 mL of 9 m HCl aqueous, followed by stirring for 10 min. Subsequently, 0.5 g of Ti_3_AlC_2_ was slowly added into the above etching solution at room temperature and kept stirring for 24 h. The acidic mixture was repeatedly washed with deionized H_2_O and centrifuged until the pH was ≈6. A stable dark green supernatant could be observed after centrifugation at 3500 rpm for 1 h. Finally, the resulting suspension was freeze‐dried for 2 days to obtain Ti_3_C_2_T*_x_* nanosheets.

### Synthesis of Ti_3_C_2_T*_x_*/g‐C_3_N_4_ Hybrid

Ti_3_C_2_T*_x_*/g‐C_3_N_4_ hybrid was prepared through the processes of self‐assembly and in situ calcination reaction. Typically, a certain amount of DCD was added into 50 mL of Ti_3_C_2_T*_x_* aqueous (2 mg mL^−1^), followed by sonicating for 1 h to obtain a homogeneous solution. The collected solution was freeze‐dried for 2 days to obtain Ti_3_C_2_T*_x_*/DCD composite. DCD monomers were deposited on the Ti_3_C_2_T*_x_* surface through weak interaction (e.g., hydrogen bonding) during the self‐assembly process, enabling uniform distribution of DCD on the surface of Ti_3_C_2_T*_x_*. The Ti_3_C_2_T*_x_*/DCD composite was then annealed at 550 °C (ramping rate of 5 °C min^−1^) for 2 h under Ar atmosphere. During this process, DCD was in situ condensed into g‐C_3_N_4_ on the surface of Ti_3_C_2_T*_x_*, obtaining the Ti_3_C_2_T*_x_*/g‐C_3_N_4_ hybrid. The Ti_3_C_2_T*_x_*/g‐C_3_N_4_ hybrid was optimized by tuning the addition amount of DCD from 0.3, 0.5, 0.7 to 0.9 g, which was denoted as Ti_3_C_2_T*_x_*/g‐C_3_N_4_‐3, Ti_3_C_2_T*_x_*/g‐C_3_N_4_‐5, Ti_3_C_2_T*_x_*/g‐C_3_N_4_‐7, and Ti_3_C_2_T*_x_*/g‐C_3_N_4_‐9, respectively. Besides, pure g‐C_3_N_4_ was synthesized via direct calcination of DCD with the same procedure.

### Structural Characterization

The morphologies and elemental components of the samples were characterized by SEM (Hitachi SU8020) and TEM (JEOL JEM‐2100F) equipped with energy dispersive spectrometry (EDS) at an acceleration voltage of 200 kV, respectively. The XRD patterns were recorded on an X‐ray diffractometer at 40 kV and 40 mA using Cu K*α* radiation (Bruker D8 Advance). XPS spectra were collected on the ESCALAB 250Xi, (Thermo Fisher Scientific Inc., USA) equipped with Ar ion etching (2 kV; 2 µA). To obtain Brunauer–Emmett–Teller (BET) specific surface areas of the materials, nitrogen adsorption–desorption isotherms were recorded by nitrogen adsorption apparatus (ASAP2020, Micromeritics). FTIR characterization was carried out on a Bruker Vertex V70 spectrometer in the diffuse reflection mode with a Spectra Tech Collector II accessory. Depth profiling and chemical analysis data of the sample were collected on a TOF‐SIMS instrument (IONTOF GmbH, Germany 2010). The data were recorded in ultrahigh vacuum at a pressure of 10^−9^ Torr in a negative model.

### Electrochemical Measurements

The galvanostatic charge–discharge experiments were carried out using battery testing system (Neware BTS 4000) at room temperature. For half cells, CR2025‐type coin cells (MTI Corporation) were assembled with Li foil as the counter electrode, Ti_3_C_2_T*_x_*/g‐C_3_N_4_ electrode (Ti_3_C_2_T*_x_* or g‐C_3_N_4_ electrode) as the working electrode to investigate the process of Li plating/stripping. All the coin cells were assembled in an argon‐filled glovebox. One piece of separator (Celgard 2400, 25 µm thickness) was used to separate Li foil (150 µm thickness) and the working electrode. 40 µL of 1.0 m lithium bis(trifluoromethanesulfonyl)imide (LiTFSI) in 1,3‐dioxolane (DOL)/1,2‐dimethoxyethane (DME) mixed solution (1:1 by volume, with 1.0 wt% of LiNO_3_ as the additive) was used as the electrolyte. Working electrodes were prepared through the typical slurry‐making process. Active electrode materials (Ti_3_C_2_T*_x_*/g‐C_3_N_4_ hybrid or pure Ti_3_C_2_T*_x_*), carbon black (super P C65), and polyvinylidene difluoride (PVDF) were mixed homogeneously with a weight ratio of 8:1:1, stirring in *N*‐methyl‐2‐pyrrolidone (NMP) solvent for 3 h to form uniform slurries. The slurries were then pasted onto clean copper foil collector through doctor blading, followed by drying at 85 °C overnight in a vacuum oven. All the current collectors were cut into round pieces (about 1.12 cm^2^). Cycling tests were performed by depositing different capacities of Li on the working electrodes, and then charging to 1.0 V (vs Li^+^/Li) for each cycle at different current densities. The batteries were first cycled between 0.01 and 1.0 V at 0.05 mA for five cycles to stabilize the surface state of the electrodes. The EIS were carried out on the electrochemical workstation (CHI 760E) with the frequency changing from 0.01 Hz to 100 kHz.

For symmetrical cells, 1.0 mAh cm^−2^ of Li was electrodeposited onto the Ti_3_C_2_T*_x_*/g‐C_3_N_4_ electrodes (or Ti_3_C_2_T*_x_* electrodes) at a current density of 0.5 mA cm^−2^, which were then disassembled from the half cells to obtain Li@Ti_3_C_2_T*_x_*/g‐C_3_N_4_ electrodes (or Li@Ti_3_C_2_T*_x_* electrodes). Two pieces of Li@Ti_3_C_2_T*_x_*/g‐C_3_N_4_ electrodes (or Li@Ti_3_C_2_T*_x_* electrodes) were used to assemble a symmetrical cell. Symmetrical cells were tested at 0.5 mAh cm^−2^ at 0.5 mA cm^−2^.

The full cells were assembled with 40 µL of electrolyte, commercial LFP as the cathodes, and Li@Ti_3_C_2_T*_x_*/g‐C_3_N_4_ (or Li@Ti_3_C_2_T*_x_*) as anodes. The LFP cathodes were prepared by casting NMP slurry containing LFP, Super P, and PVDF with a weight ratio of 8:1:1 onto Al foil, followed by drying at 85 °C overnight in a vacuum oven. Before matching the full cells, LFP cathodes were first cycled several times paired with Li anodes to stabilize the cathode electrolyte interphase. In addition, a certain amount of Li was electrodeposited onto the Ti_3_C_2_T*_x_*/g‐C_3_N_4_ electrodes (or Ti_3_C_2_T*_x_* electrodes) to obtain Li@Ti_3_C_2_T*_x_*/g‐C_3_N_4_ anodes (or Li@Ti_3_C_2_T*_x_* anodes), which were then disassembled from the coin cells and utilized for assembling the full cells. The full cells were cycled within the voltage range of 2.2 to 4.0 V (vs Li/Li^+^).

When paired with 0.64 mAh cm^−2^ LFP, 1.6 mAh cm^−2^ of Li was electrodeposited onto the Ti_3_C_2_T*_x_*/g‐C_3_N_4_ electrodes (or Ti_3_C_2_T*_x_* electrodes) to obtain Li@Ti_3_C_2_T*_x_*/g‐C_3_N_4_ anodes (or Li@Ti_3_C_2_T*_x_* anodes), which were then disassembled from the coin cells and utilized for assembling the full cells. The capacity ratio of negative electrode to the positive electrode (N/P) was 2.5.

When paired with 0.64 mAh cm^−2^ LFP, 0.32 mAh cm^−2^ of Li was electrodeposited onto the Ti_3_C_2_T*_x_*/g‐C_3_N_4_ electrodes (or Ti_3_C_2_T*_x_* electrodes) to obtain Li@Ti_3_C_2_T*_x_*/g‐C_3_N_4_ anodes (or Li@Ti_3_C_2_T*_x_* anodes), which were then disassembled from the coin cells and utilized for assembling the full cells. The capacity ratio of negative electrode to the positive electrode (N/P) was 0.5.

When paired with 2.2 mAh cm^−2^ LFP, 2.2 mAh cm^−2^ of Li was electrodeposited onto the Ti_3_C_2_T*_x_*/g‐C_3_N_4_ electrodes (or Ti_3_C_2_T*_x_* electrodes) to obtain Li@Ti_3_C_2_T*_x_*/g‐C_3_N_4_ anodes (or Li@Ti_3_C_2_T*_x_* anodes), which were then disassembled from the coin cells and utilized for assembling the full cells. The capacity ratio of negative electrode to the positive electrode (N/P) was 1.0.

In addition, the full cells were assembled with 40 µL of electrolyte (1.0 m LiPF_6_ in EC/DMC, 1:1 by volume), LiNi_0.8_Co_0.1_Mn_0.1_O_2_ (NCM811) as the cathodes, and Li@Ti_3_C_2_T*_x_*/g‐C_3_N_4_ (or Li@Ti_3_C_2_T*_x_*) as anodes. When paired with 1.2 mAh cm^−2^ NCM811, 3.0 mAh cm^−2^ of Li was electrodeposited onto the Ti_3_C_2_T*_x_*/g‐C_3_N_4_ electrodes (or Ti_3_C_2_T*_x_* electrodes) to obtain Li@Ti_3_C_2_T*_x_*/g‐C_3_N_4_ anodes (or Li@Ti_3_C_2_T*_x_* anodes), which were then disassembled from the coin cells and utilized for assembling the full cells. The full cells coupled with NCM811 were cycled within the voltage range of 2.7 to 4.4 V (vs Li/Li^+^). The capacity ratio of negative electrode to the positive electrode (N/P) was 2.5.

## Conflict of Interest

The authors declare no conflict of interest.

## Supporting information

Supporting Information

## Data Availability

Research data are not shared.
