# Supplementary material for: Constructing Artificial SEI Layer on Lithiophilic MXene Surface for High‐Performance Lithium Metal Anodes
Source: Adv Sci (Weinh). 2022 Jan 6;9(6):2103930. doi: 10.1002/advs.202103930 (PMC8867166; doi:10.1002/advs.202103930)
Supplement: Supplementary file 1 — Supporting Information [file ADVS-9-2103930-s001.pdf]

ADVANCED SCIENCE
Open Access

Supporting Information

for *Adv. Sci.*, DOI: 10.1002/advs.202103930

Constructing Artificial SEI Layer on Lithiophilic MXene Surface for High-Performance Lithium Metal Anodes

*Feifei Zhao, Pengbo Zhai, Yi Wei, Zhilin Yang, Qian Chen, Jinghan Zuo, Xiaokang Gu, and Yongji Gong**

Supporting Information

Constructing Artificial SEI Layer on Lithiophilic MXene Surface for High-Performance Lithium Metal Anodes

*Feifei Zhao, Pengbo Zhai, Yi Wei, Zhilin Yang, Qian Chen, Jinghan Zuo, Xiaokang Gu, and Yongji Gong**

Dr. F. Zhao, Dr. P. Zhai, Z. Yang, Q. Chen, J. Zuo, X. Gu, Prof. Y. Gong
School of Materials Science and Engineering, Beihang University, Beijing 100191, China
E-mail: yongjigong@buaa.edu.cn

Dr. P. Zhai
College of Physics, Qingdao University, Qingdao 266071, China

Y. Wei
Beijing Key Laboratory of Electrochemical Process and Technology for Materials, Beijing University of Chemical Technology, Beijing 100029, China

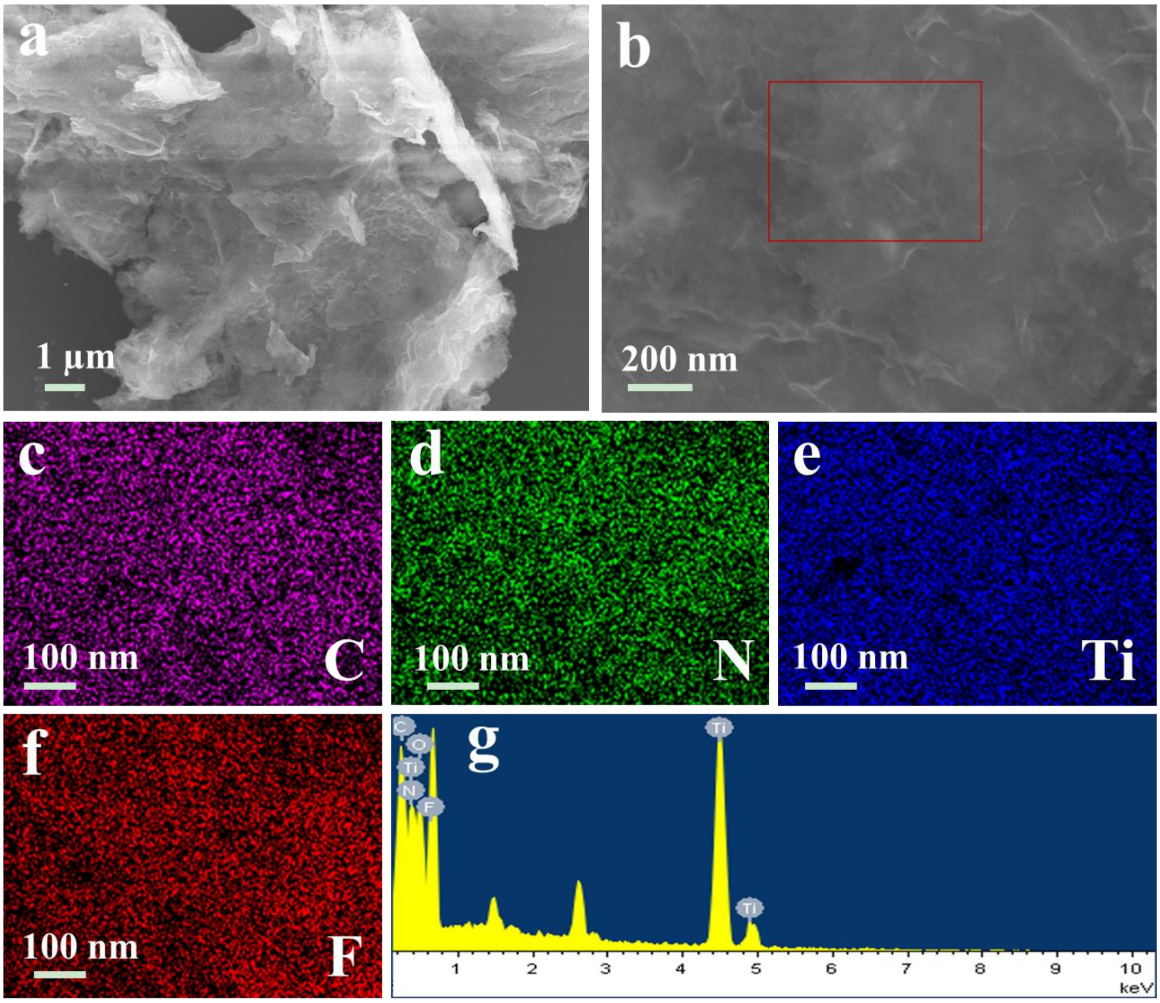

Figure S1. (a,b) SEM images of freeze-drying $Ti_3C_2T_x$/DCD, (c-f) the corresponding elemental mappings and (g) EDS spectrum of $Ti_3C_2T_x$/DCD, indicating uniform distribution of DCD on the $Ti_3C_2T_x$ surface without destroying the nanosheet morphology of $Ti_3C_2T_x$.

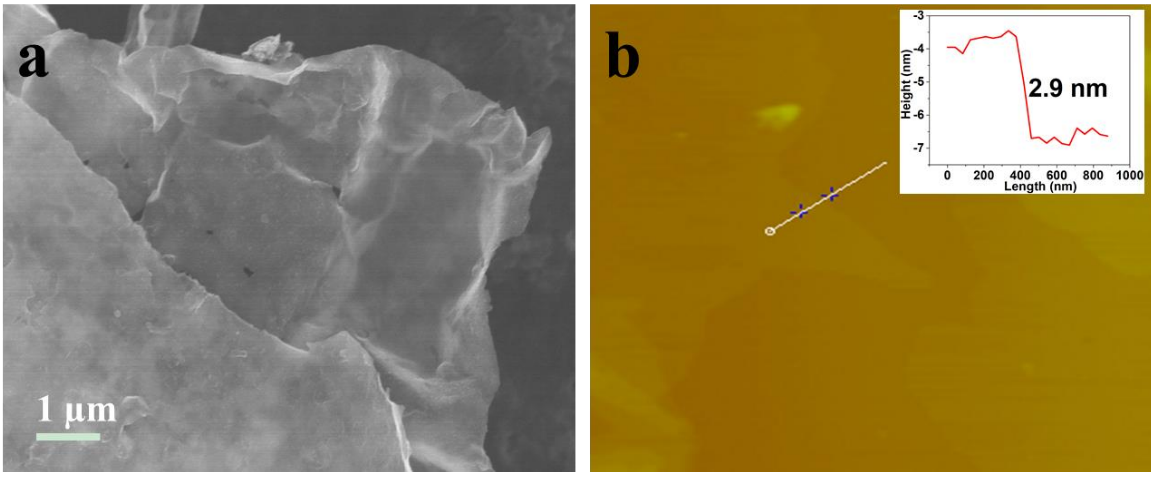

Figure S2. (a) SEM and (b) AFM images of bare $Ti_3C_2T_x$.

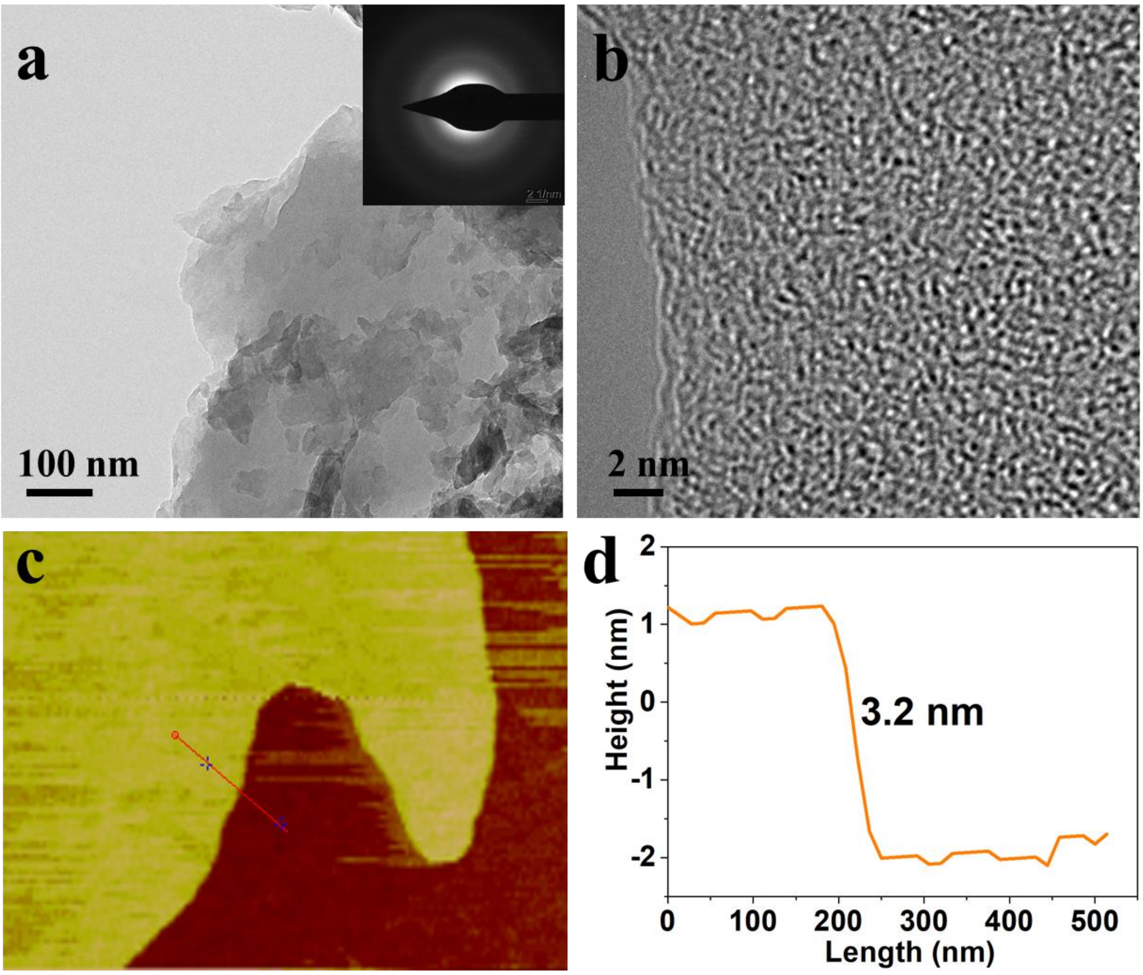

Figure S3. (a) TEM , (b) HRTEM , (c) AFM images, and (d) the corresponding height profile of pristine g-C_3N_4, indicating the nanosheet morphology. Inset in (a) shows the corresponding SAED pattern.

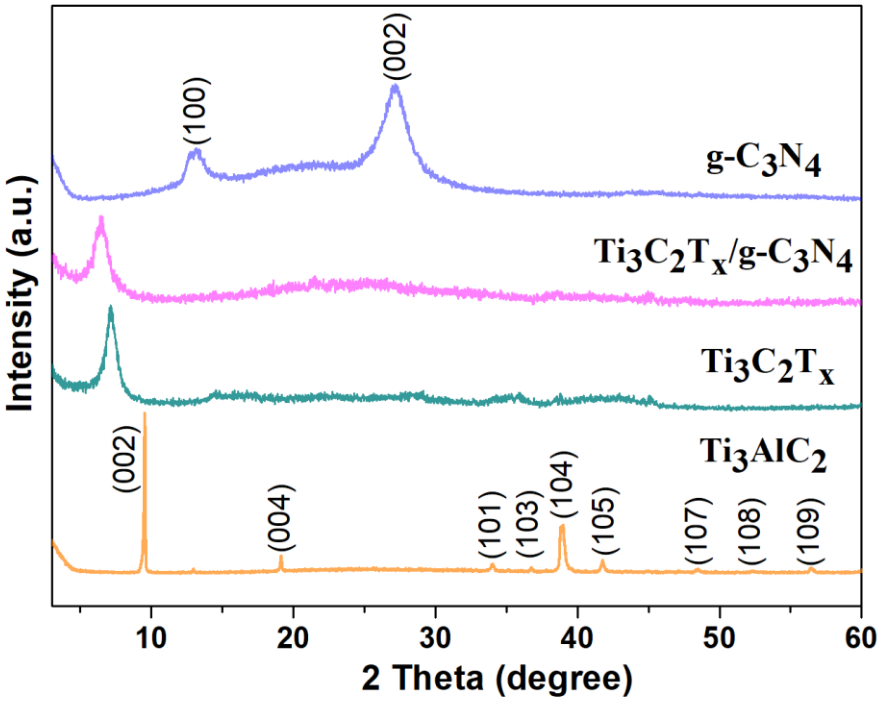

Figure S4. XRD patterns of $Ti_3C_2T_x$, g-C_3N_4, and $Ti_3C_2T_x$/g-C_3N_4 hybrid.

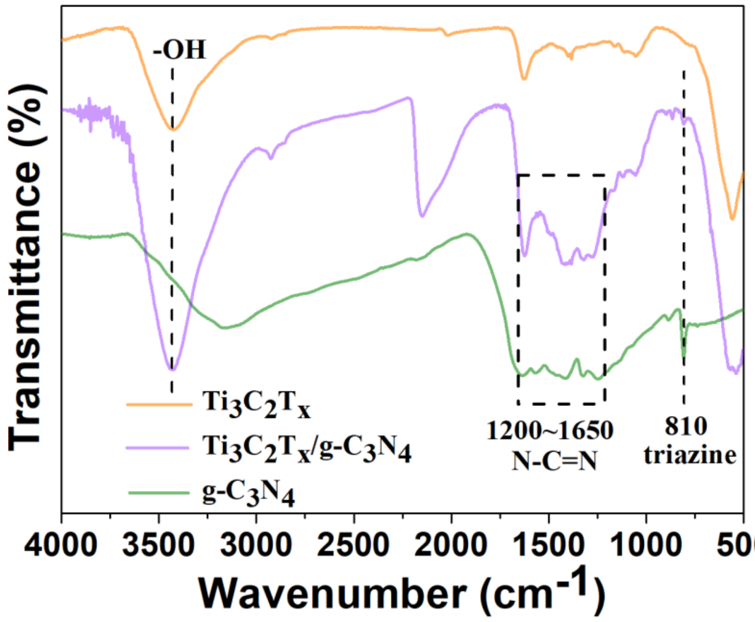

Figure S5. FT-IR spectra of $Ti_3C_2T_x$, g-C_3N_4, and $Ti_3C_2T_x$/g-C_3N_4 hybrid.

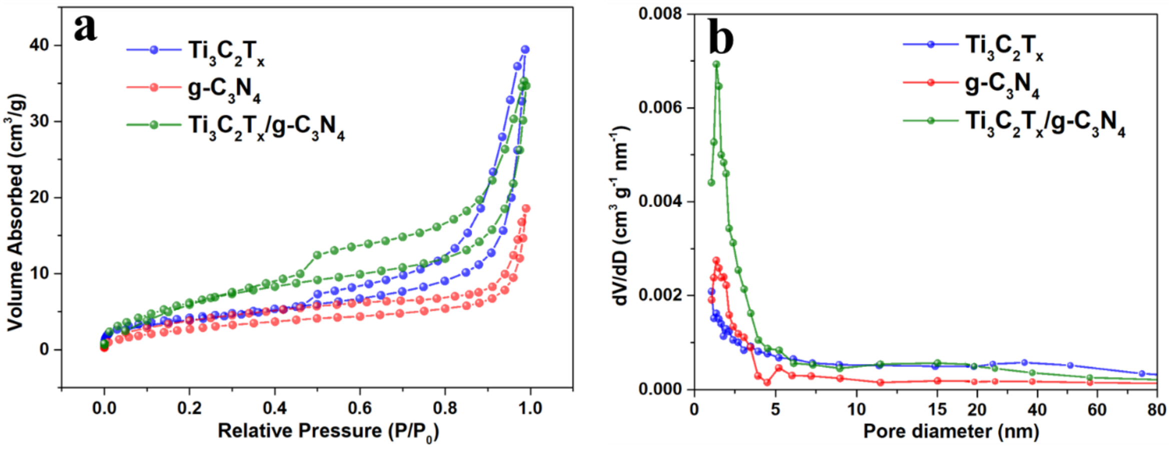

Figure S6. (a) N_2 adsorption-desorption isotherms and (b) the pore diameter distributions of $Ti_3C_2T_x$, g-C_3N_4, and $Ti_3C_2T_x$/g-C_3N_4 hybrid.

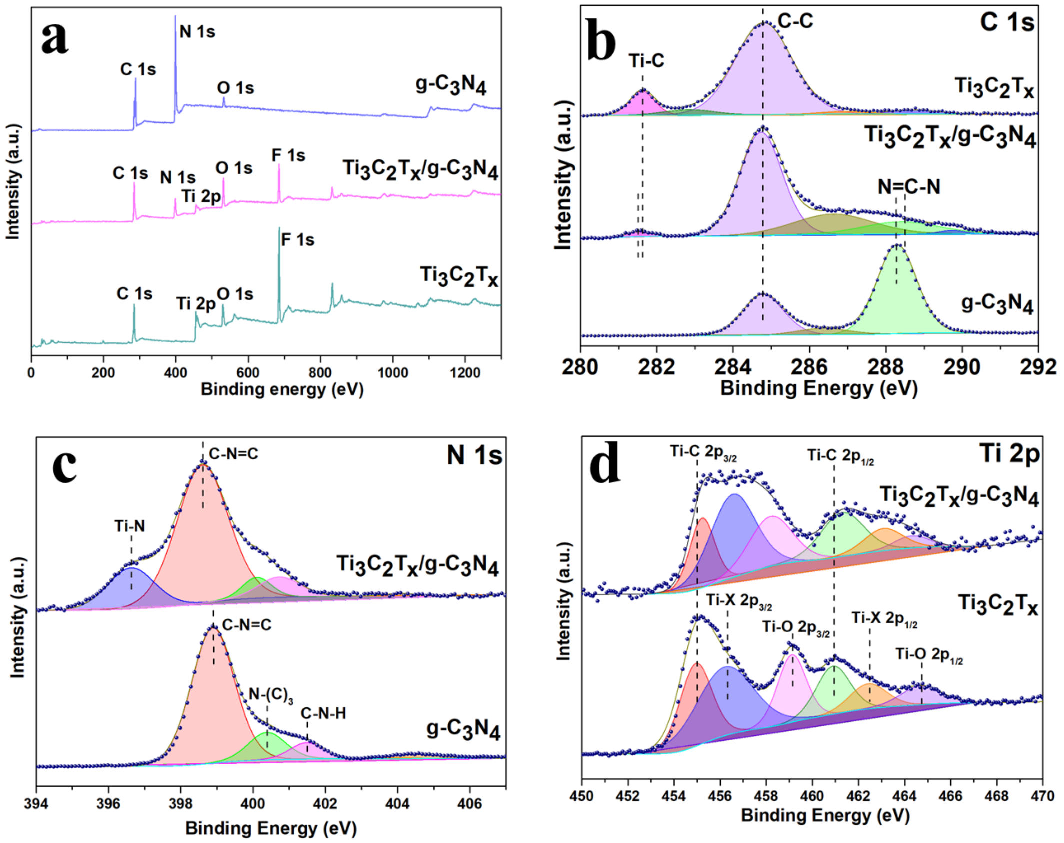

Figure S7. XPS spectra of $Ti_3C_2T_x$, $g\text{-}C_3N_4$, and $Ti_3C_2T_x/g\text{-}C_3N_4$ hybrid: (a) full spectra, high-resolution spectra of (b) C 1s, (c) N 1s, and (d) Ti 2p.

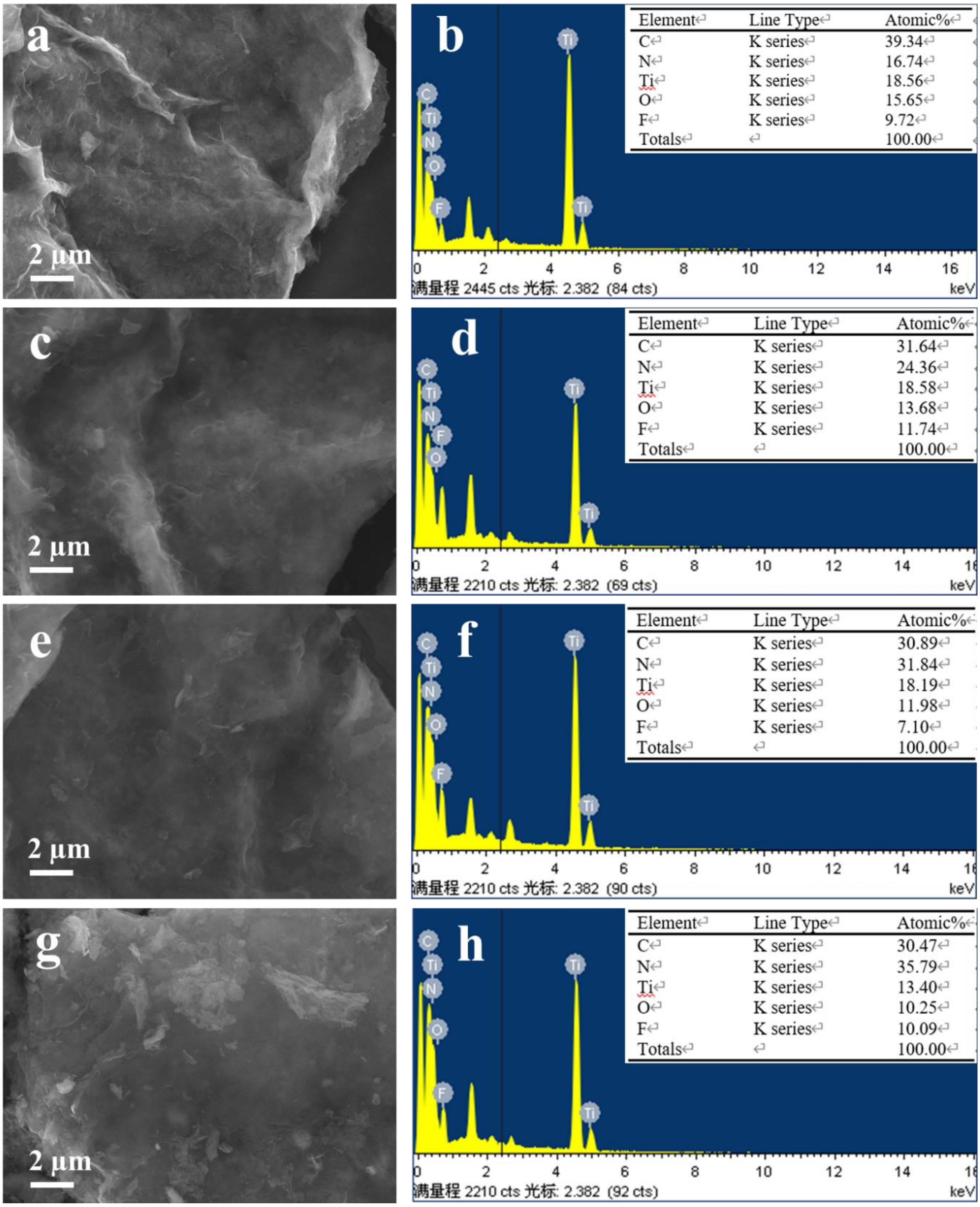

Figure S8. SEM images and EDS spectra of $Ti_3C_2T_x/g\text{-}C_3N_4$ composites with diffferent $g\text{-}C_3N_4$ content. (a) SEM image and (b) EDS spectrum of $Ti_3C_2T_x/g\text{-}C_3N_4\text{-}3$. (c) SEM image and

(d) EDS spectrum of $Ti_3C_2T_x$/g-C_3N_4-5. (e) SEM image and (f) EDS spectrum of $Ti_3C_2T_x$/g-C_3N_4-7. (g) SEM image and (h) EDS spectrum of $Ti_3C_2T_x$/g-C_3N_4-9.

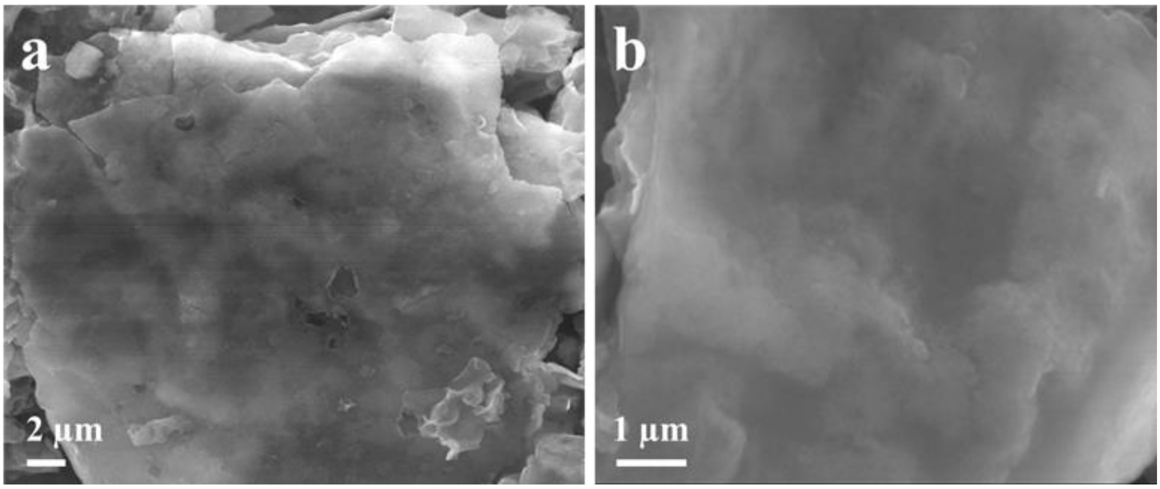

Figure S9. SEM images of $Ti_3C_2T_x$/g-C_3N_4 electrode after stripping 3 mAh/cm^2 of Li at 0.5 mA/cm^2.

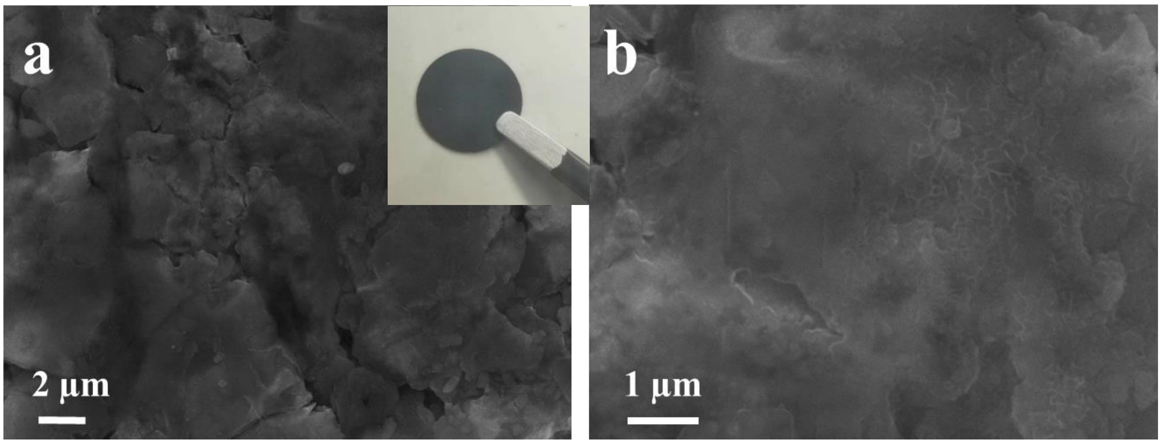

Figure S10. SEM images of $Ti_3C_2T_x$/g-C_3N_4 electrode after 50 cycles of Li plating/stripping with an areal capacity of 1 mAh cm^{-2} at 1.0 mA cm^{-2}. The inset image in (a) is photograph of the corresponding electrode.

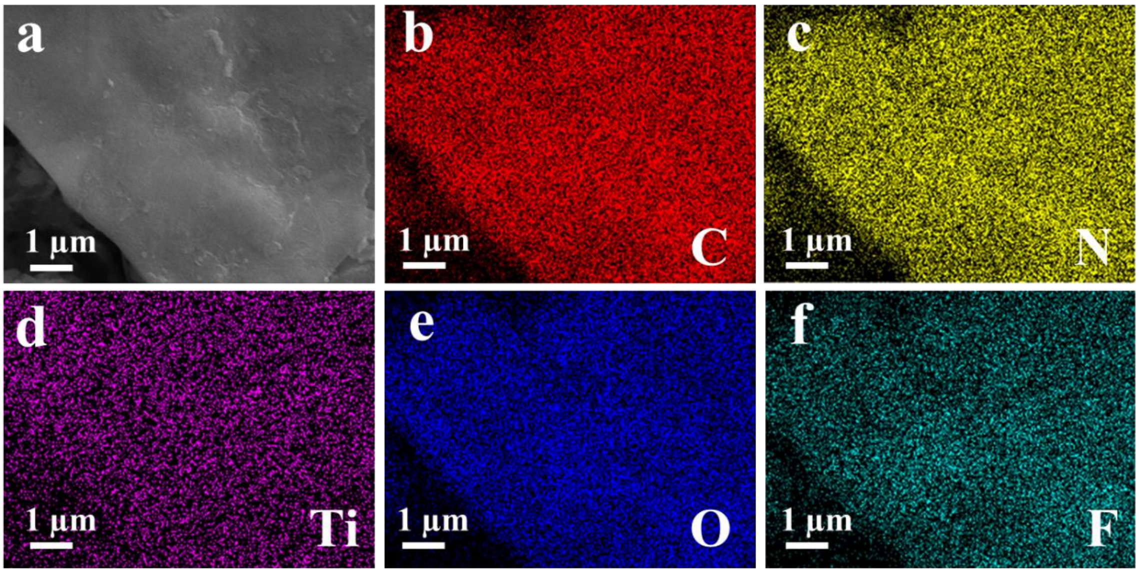

Figure S11. (a) SEM images of $Ti_3C_2T_x$/g-C_3N_4 electrode after 50 cycles of Li plating/stripping with an areal capacity of 1 mAh cm^{-2} at 1.0 mA cm^{-2}. (b-f) The corresponding elemental mappings.

According to the corresponding elemental mappings of $Ti_3C_2T_x$/g-C_3N_4 electrode after 50 cycles, the C, N, Ti, O, and F elements homogeneously distribute over the sample without element accumulates. The $Ti_3C_2T_x$/g-C_3N_4 retains the original overall morphology, indicating the good structure stability ensured by the artificial SEI layer.

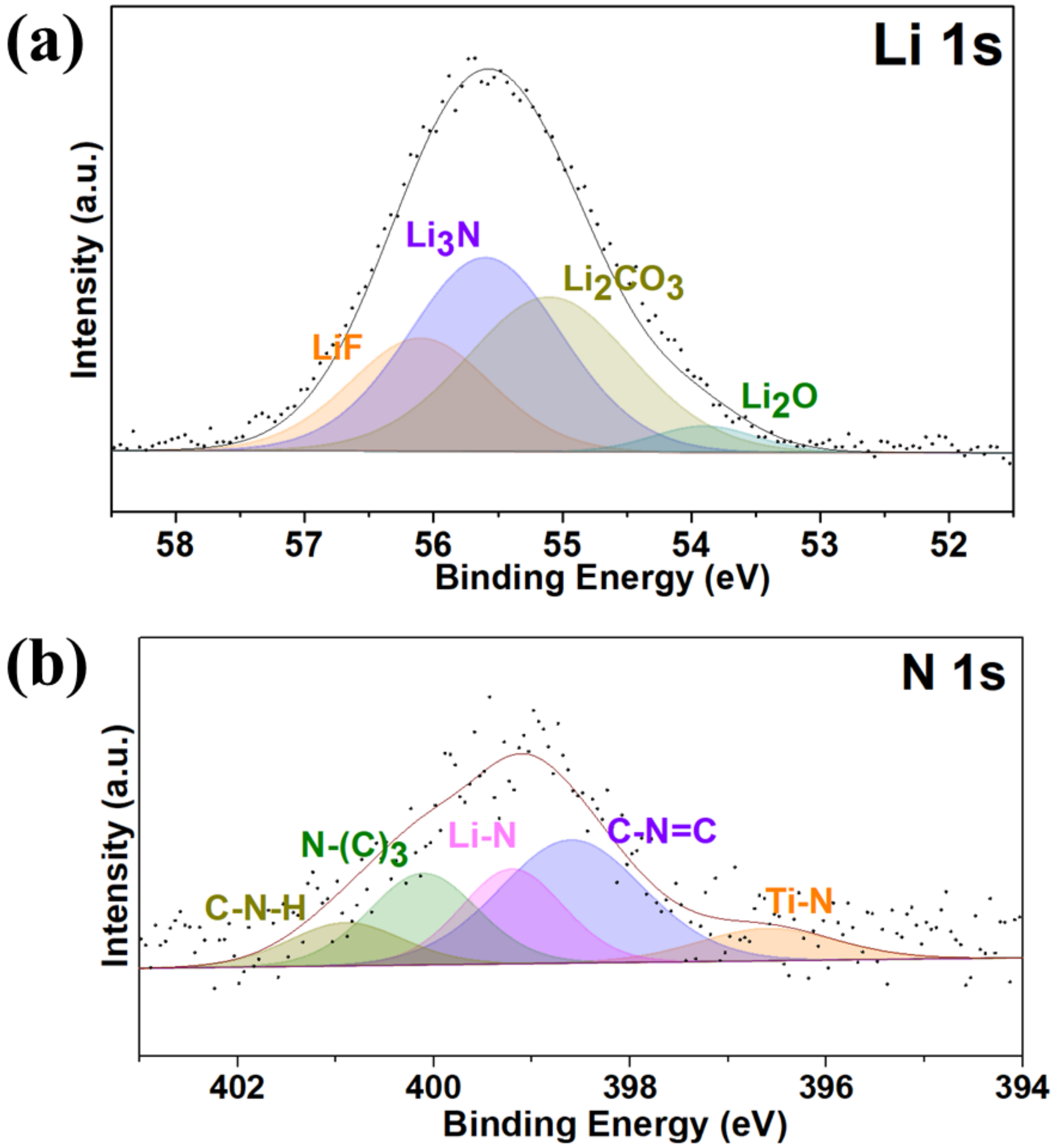

Figure S12. High-resolution (a) Li 1s and (b) N 1s XPS spectra of $Ti_3C_2T_x$/g-C_3N_4 electrode after 50 cycles of Li plating/stripping with an areal capacity of 1 mAh cm^{-2} at 1.0 mA cm^{-2}.

In the N 1s XPS spectra of $Ti_3C_2T_x$/g-C_3N_4 electrode after 50 cycles, the C-N=C and N-(C)$_3$ characteristic peaks of g-C_3N_4 are dominant, indicating the structure stability of g-C_3N_4 during cycling.

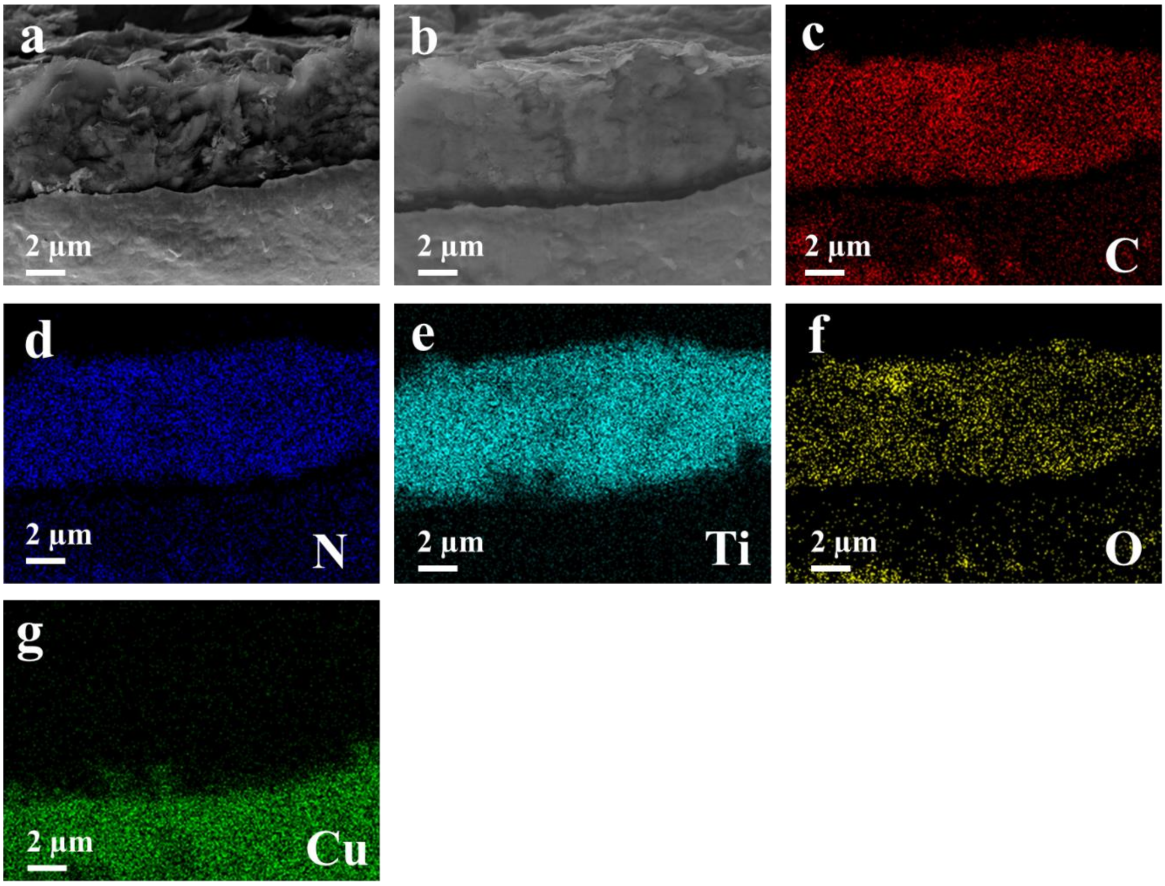

Figure S13. (a) Cross-section SEM image of the $Ti_3C_2T_x$/g-C_3N_4 electrode before Li deposition. (b) Cross-section SEM image of $Ti_3C_2T_x$/g-C_3N_4 electrode after plating 1 mAh cm^{-2} of Li, and (c-g) the corresponding elemental mappings.

In the $Ti_3C_2T_x$/g-C_3N_4 composite electrode, $Ti_3C_2T_x$ serves as a 3D conductive host and provids sufficient lithiophilic sites for Li nucleation. g-C_3N_4 functions as an artificial SEI layer constructed on the $Ti_3C_2T_x$ surface to ensure the stability of the Li/electrolyte interface. Cross-section SEM image of the $Ti_3C_2T_x$/g-C_3N_4 electrode before Li deposition shows that the original $Ti_3C_2T_x$/g-C_3N_4 possesses 3D interconnected network structure. After Li plating, the $Ti_3C_2T_x$/g-C_3N_4 electrode became more compact.

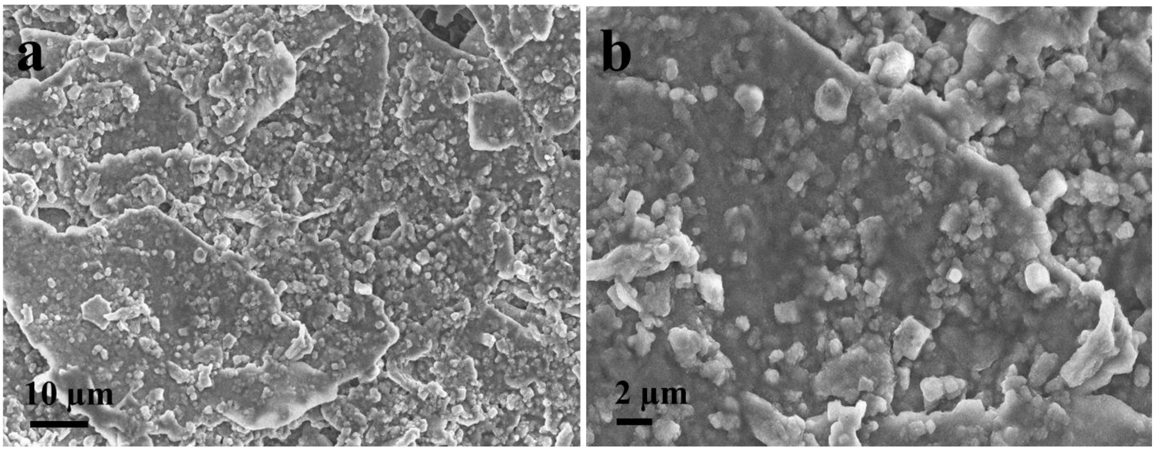

Figure S14. SEM images of $Ti_3C_2T_x$ electrode after stripping 3 mAh/cm^2 of Li at 0.5 mA/cm^2.

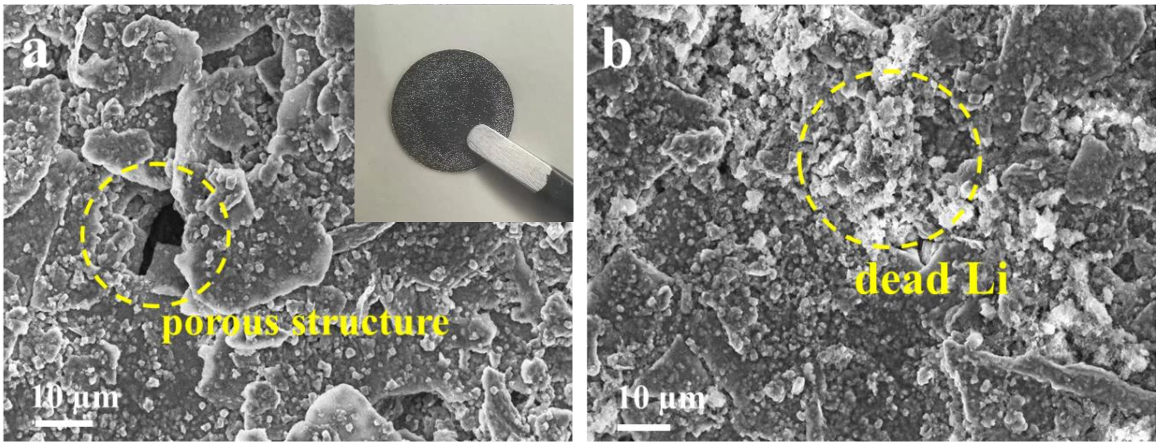

Figure S15. SEM images of $Ti_3C_2T_x$ electrode after 50 cycles of Li plating/stripping with an areal capacity of 1 mAh cm^{-2} at 1.0 mA cm^{-2}. The inset image in (a) is photograph of the corresponding electrode.

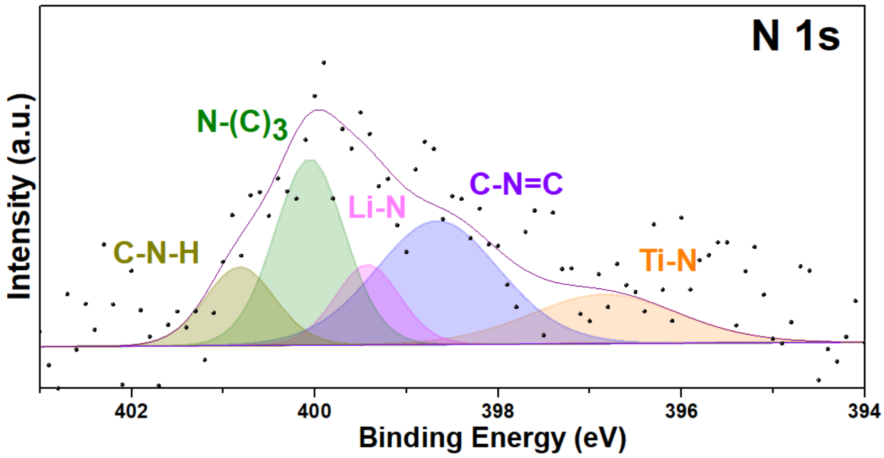

Figure S16. N 1s XPS depth profiles of $Ti_3C_2T_x$/g-C_3N_4 electrode after depositing 1.0 mAh cm^{-2} of Li at 0.5 mA cm^{-2}.

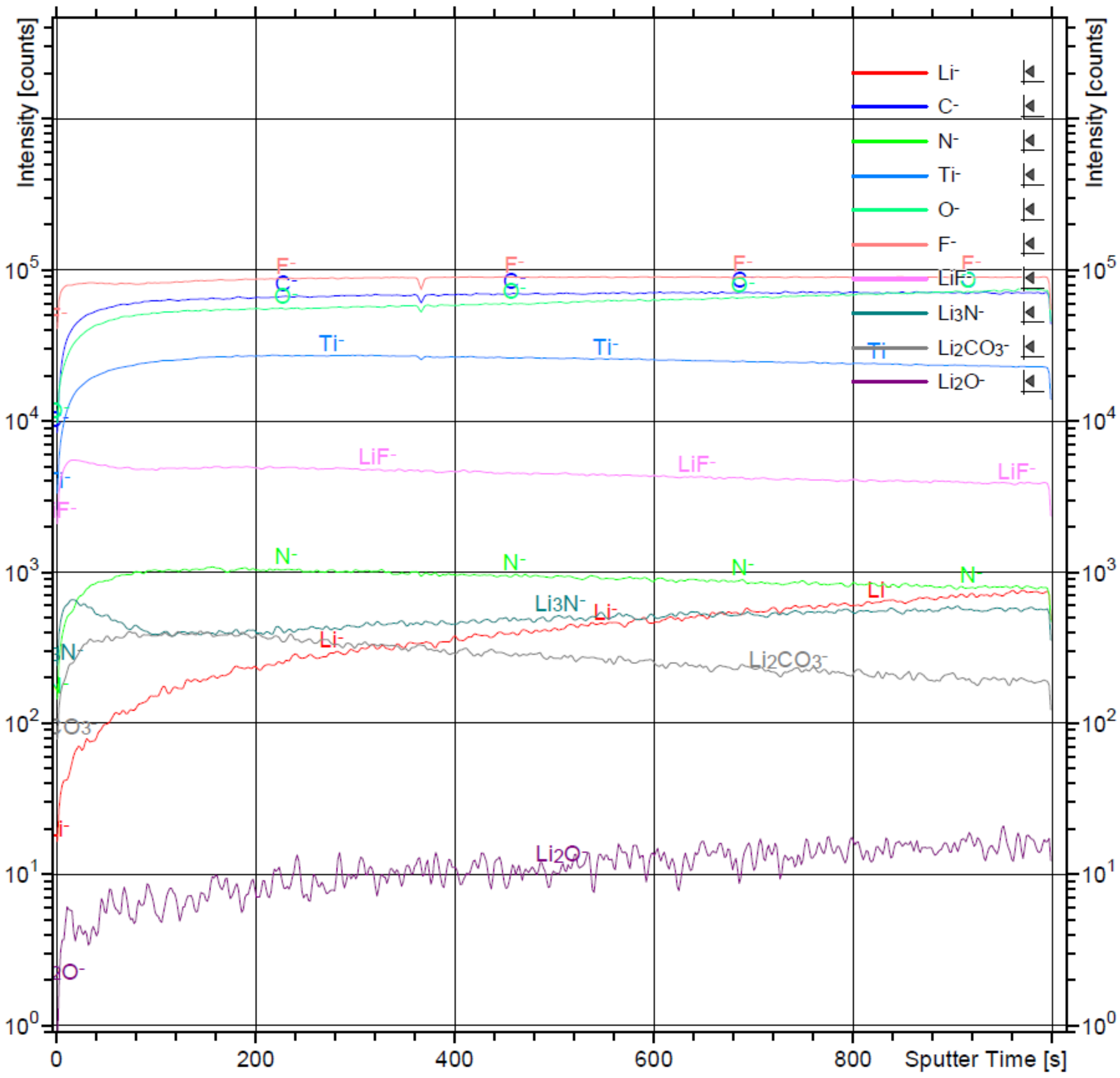

Figure S17. TOF-SIMS in-depth profiles of relevant secondary ion fragments taken at the surface of $Ti_3C_2T_x$/g-C_3N_4 electrode after depositing 1 mAh cm^{-2} of Li. The TOF-SIMS in-depth profiles reveal the evolution of several fragments as the sputtering proceeds in a negative mode.

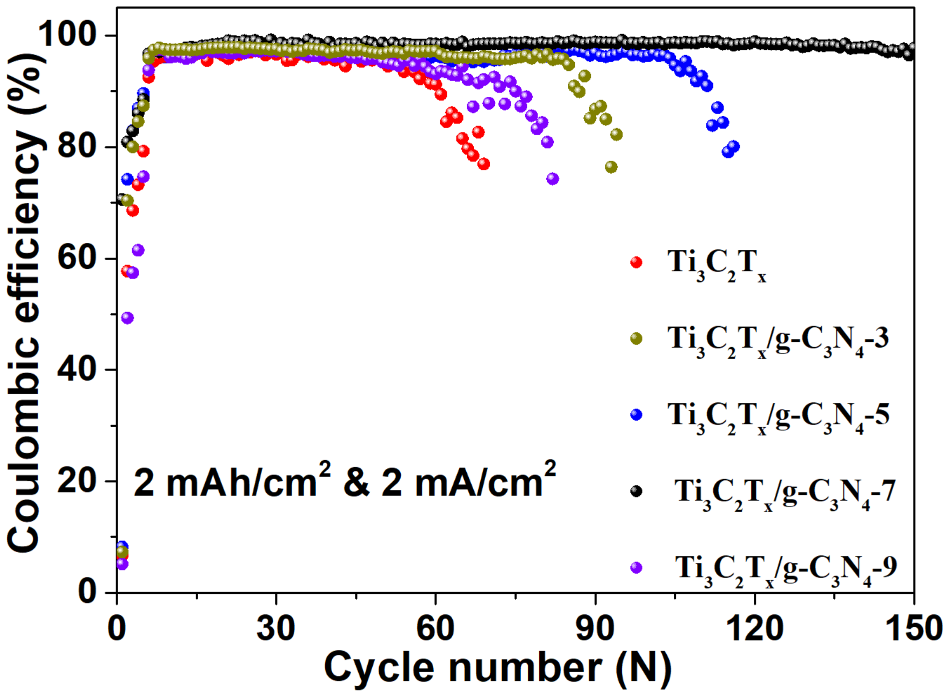

Figure S18. Coulombic efficiency of galvanostatic Li plating/stripping cycling on $Ti_3C_2T_x$ electrode and $Ti_3C_2T_x$/g-C_3N_4 composite electrodes with diffferent g-C_3N_4 content under deposition capacity of 2 mAh cm^{-2} at 2 mA cm^{-2}.

To optimize the g-C_3N_4 content in $Ti_3C_2T_x$/g-C_3N_4 composites, CE of galvanostatic Li plating/stripping cycling on different electrodes were tested. When the cycling capacity was 2 mAh cm^{-2} at a current density of 2 mA cm^{-2}, the CE of $Ti_3C_2T_x$ electrode fluctuated in the first 60 cycles and dropped down significantly afterwards, which may ascribe to the dendrites formation on the surface of $Ti_3C_2T_x$. For $Ti_3C_2T_x$/g-C_3N_4 composite electrodes, with the content of g-C_3N_4 increasing from $Ti_3C_2T_x$/g-C_3N_4-3 to $Ti_3C_2T_x$/g-C_3N_4-7, more stable cycling and longer lifespans were obtained owing to the homogeneous Li plating/stripping, which was attributed to the introduction of g-C_3N_4 served as the artificial SEI. $Ti_3C_2T_x$/g-C_3N_4-7 electrode exhibited optimum cycling stability for 150 cycles with high average CE of 98.6%. Nevertheless, further increasing the g-C_3N_4 content caused inferior cycling stability, because excessive g-C_3N_4 on the $Ti_3C_2T_x$ surface may hamper the lithiophilic sites and electron- and ion-conducting paths in $Ti_3C_2T_x$.

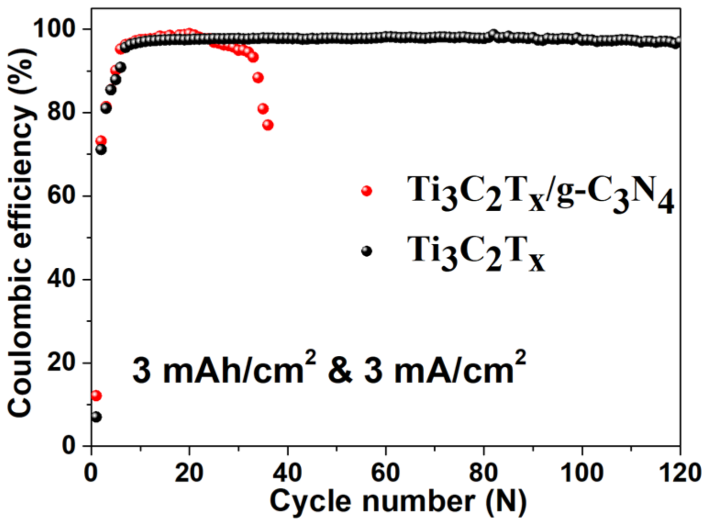

Figure S19. Coulombic efficiency of galvanostatic Li plating/stripping cycling on $Ti_3C_2T_x$/g-C_3N_4 and $Ti_3C_2T_x$ electrodes with the capacity of 3 mAh cm^{-2} at 3 mA cm^{-2}.

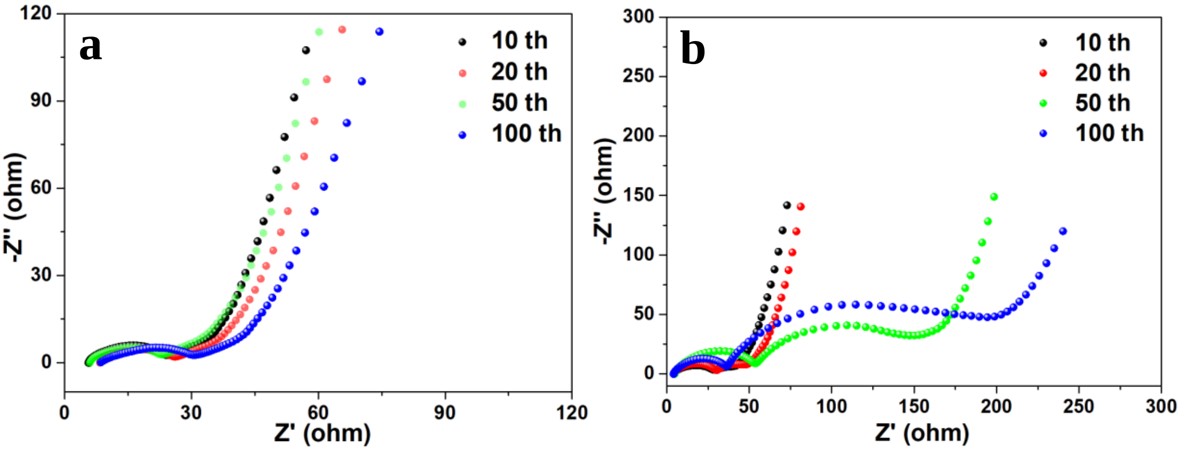

Figure S20. EIS spectra of (a) $Ti_3C_2T_x$/g-C_3N_4 and (b) $Ti_3C_2T_x$ electrodes at different cycles.

Table S1. Equivalent-circuit fitting and detailed analysis on the obtained EIS spectra of $Ti_3C_2T_x$/g-C_3N_4 and $Ti_3C_2T_x$ electrodes.

Equivalent circuit	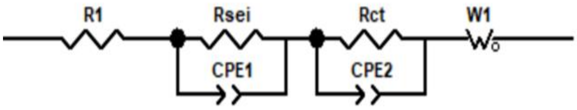		
Electrodes	Cycle number	Rsei (ohm)	Rct (ohm)
$Ti_3C_2T_x$/g-C_3N_4	10	20.06	8.74
	20	24.65	18.59
	50	16.68	15.13
	100	20.69	16.62
$Ti_3C_2T_x$	10	22.18	19.58
	20	24.7	18.94
	50	49.12	66.70
	100	31.41	117.30

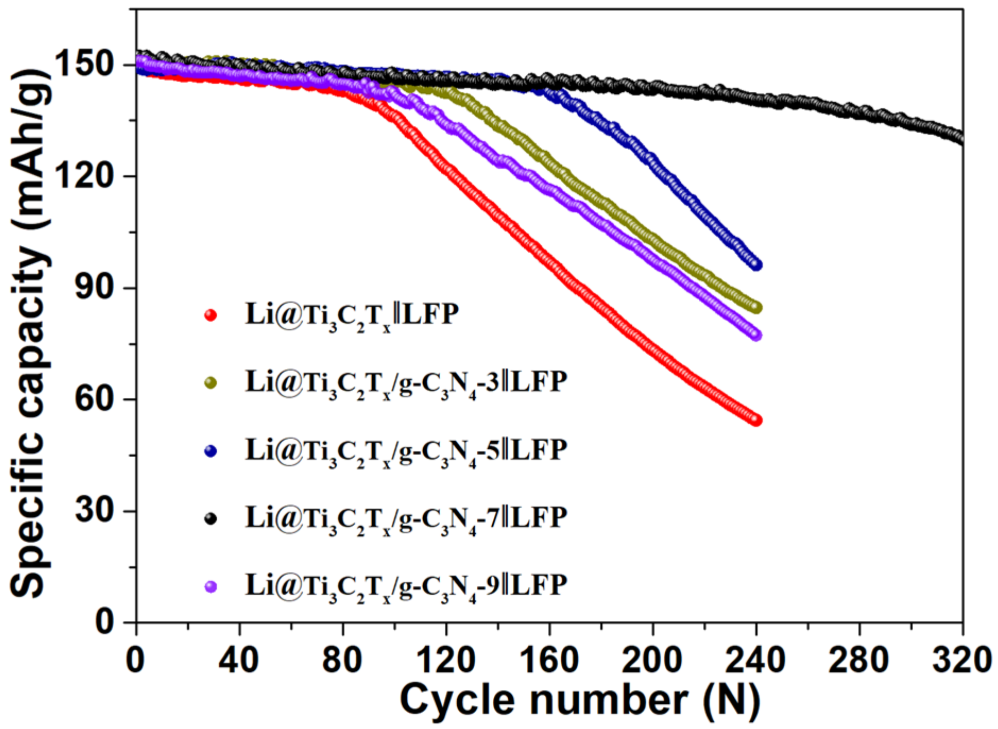

Figure S21. Electrochemical cycling performance of full cells based on $Ti_3C_2T_x$ electrode and $Ti_3C_2T_x$/g-C_3N_4 composite electrodes with diffferent g-C_3N_4 content. The full cells were cycled at 0.5 C. The areal capacity of LFP was 0.64 mAh cm^{-2} and the N/P ratio was 2.5. Compared with other full cells, the Li@$Ti_3C_2T_x$/g-C_3N_4-7||LFP full cell exhibited optimum cycling stability with highest capacity retention of 85.5% after 320 cycles at 0.5 C.

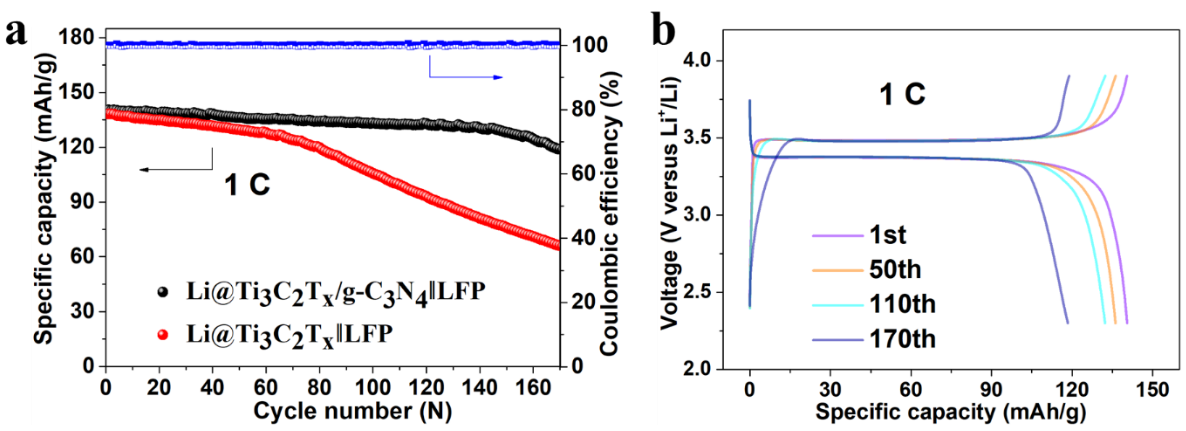

Figure S22. Electrochemical performance of Li@$Ti_3C_2T_x$/g-C_3N_4||LFP and Li@$Ti_3C_2T_x$||LFP full cells: (a) cycling performance at 1 C, (b) charge/discharge curves during cycling of

$Li@Ti_3C_2T_x/g\text{-}C_3N_4$||LFP. The areal capacity of LFP was 0.64 mAh cm^{-2} and the N/P ratio was 0.5.

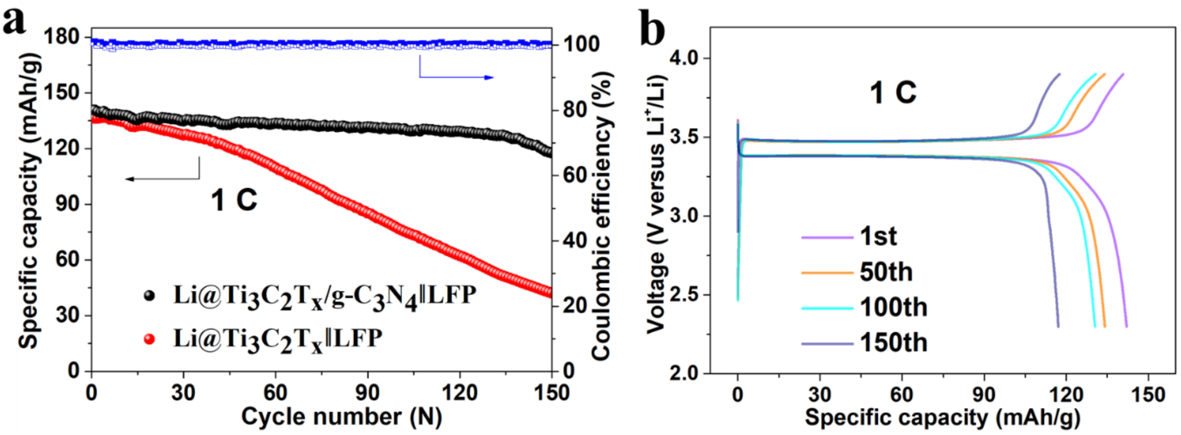

Figure S23. Electrochemical performance of $Li@Ti_3C_2T_x/g\text{-}C_3N_4$||LFP and $Li@Ti_3C_2T_x$||LFP full cells: (a) cycling performance at 1 C, (b) charge/discharge curves during cycling of $Li@Ti_3C_2T_x/g\text{-}C_3N_4$||LFP. The areal capacity of LFP was 2.2 mAh cm^{-2} and the N/P ratio was 1.

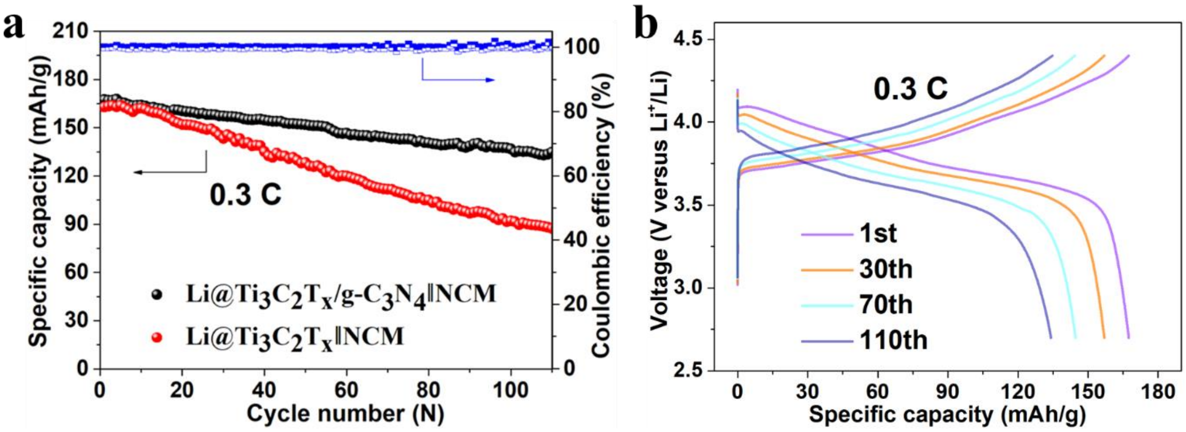

Figure S24. Electrochemical performance of $Li@Ti_3C_2T_x/g\text{-}C_3N_4$||NCM and $Li@Ti_3C_2T_x$||NCM full cells: (a) cycling performance at 0.3 C, (b) charge/discharge curves during cycling of $Li@Ti_3C_2T_x/g\text{-}C_3N_4$||NCM. The areal capacity of NCM was 1.2 mAh cm^{-2} and the N/P ratio was 2.5.

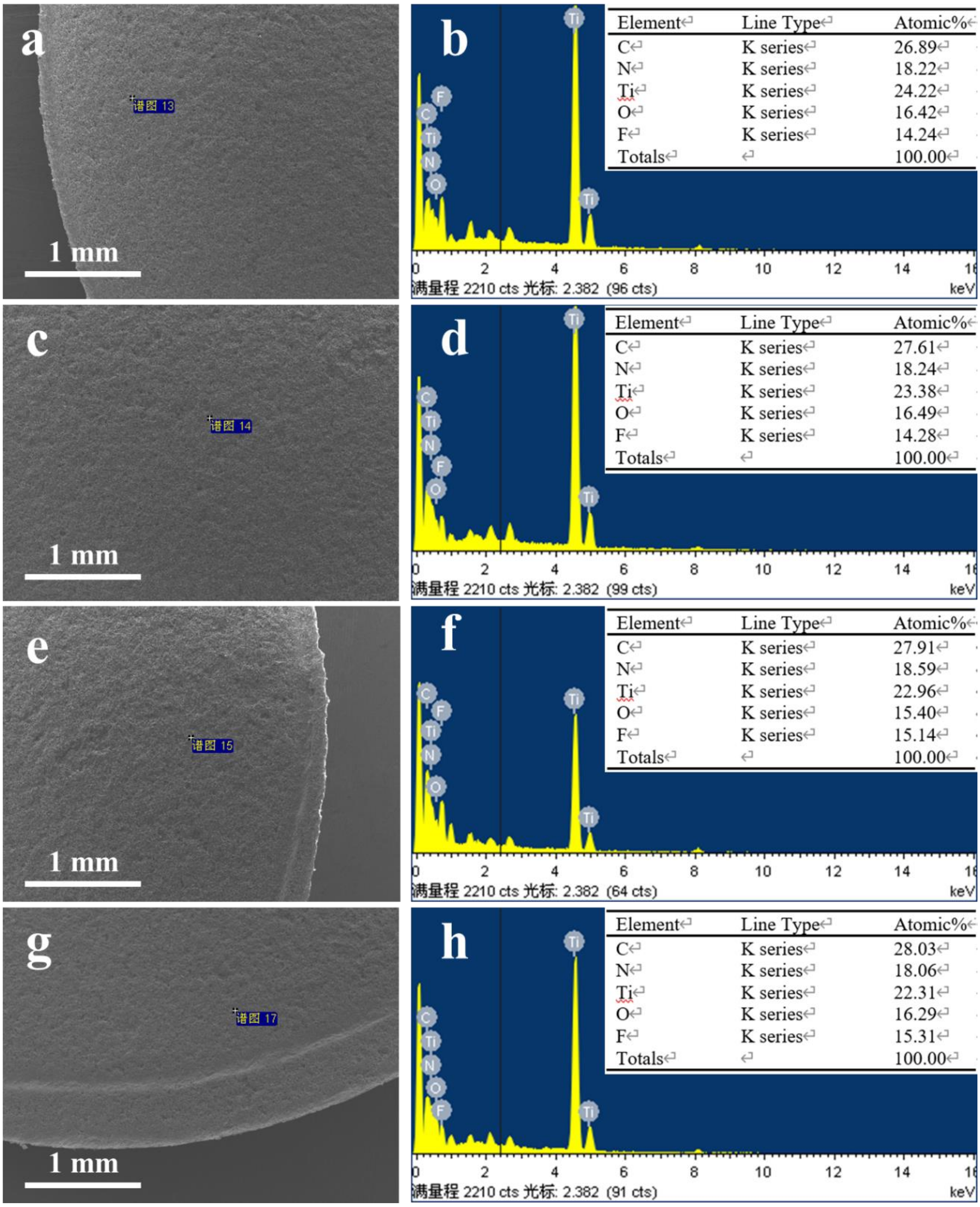

Figure S25. (a,c,e,g) SEM images of $Ti_3C_2T_x$/g-C_3N_4 electrode. (b,d,f,h) EDS spectra of four regions selected randomly on the $Ti_3C_2T_x$/g-C_3N_4 electrode.

Chemical compositions in the $Ti_3C_2T_x$/g-C_3N_4 electrode were further elucidated using EDS measurement. As shown in Figure S25, four regions are selected randomly on the $Ti_3C_2T_x$/g-C_3N_4 electrode. According to EDS analysis, the nitrogen contents in the five regions selected

randomly are very close to each other. These results confirm that g-C_3N_4 is uniformly coated on the surface of $Ti_3C_2T_x$, and the copper foil current collector is modified fully by g-C_3N_4.
